# Cytomegalovirus vectors expressing *Plasmodium knowlesi* antigens induce immune responses that delay parasitemia upon sporozoite challenge

**DOI:** 10.1371/journal.pone.0210252

**Published:** 2019-01-23

**Authors:** Scott G. Hansen, Jennie Womack, Isabel Scholz, Andrea Renner, Kimberly A. Edgel, Guangwu Xu, Julia C. Ford, Mikayla Grey, Brandyce St. Laurent, John M. Turner, Shannon Planer, Al W. Legasse, Thomas L. Richie, Joao C. Aguiar, Michael K. Axthelm, Eileen D. Villasante, Walter Weiss, Paul T. Edlefsen, Louis J. Picker, Klaus Früh

**Affiliations:** 1 Oregon Health & Science University, Vaccine & Gene Therapy Institute, Beaverton, OR, United States of America; 2 Oregon Health & Science University, Oregon National Primate Research Center, Beaverton, OR, United States of America; 3 US Military Malaria Vaccine Program, Naval Medical Research Center, Silver Spring, MD, United States of America; 4 National Institutes of Health, Laboratory of Malaria and Vector Research, Malaria Pathogenesis and Human Immunity Unit, Rockville, MD, United States of America; 5 Statistical Center for HIV/AIDS Research and Prevention, Vaccine and Infectious Disease Division, Fred Hutchinson Cancer Research Center, Seattle, WA, United States of America; University of St Andrews, UNITED KINGDOM

## Abstract

The development of a sterilizing vaccine against malaria remains one of the highest priorities for global health research. While sporozoite vaccines targeting the pre-erythrocytic stage show great promise, it has not been possible to maintain efficacy long-term, likely due to an inability of these vaccines to maintain effector memory T cell responses in the liver. Vaccines based on human cytomegalovirus (HCMV) might overcome this limitation since vectors based on rhesus CMV (RhCMV), the homologous virus in rhesus macaques (RM), elicit and indefinitely maintain high frequency, non-exhausted effector memory T cells in extralymphoid tissues, including the liver. Moreover, RhCMV strain 68–1 elicits CD8+ T cells broadly recognizing unconventional epitopes exclusively restricted by MHC-II and MHC-E. To evaluate the potential of these unique immune responses to protect against malaria, we expressed four *Plasmodium knowlesi* (Pk) antigens (CSP, AMA1, SSP2/TRAP, MSP1c) in RhCMV 68–1 or in Rh189-deleted 68–1, which additionally elicits canonical MHC-Ia-restricted CD8+ T cells. Upon inoculation of RM with either of these Pk Ag expressing RhCMV vaccines, we obtained T cell responses to each of the four Pk antigens. Upon challenge with Pk sporozoites we observed a delayed appearance of blood stage parasites in vaccinated RM consistent with a 75–80% reduction of parasite release from the liver. Moreover, the Rh189-deleted RhCMV/Pk vectors elicited sterile protection in one RM. Once in the blood, parasite growth was not affected. In contrast to T cell responses induced by Pk infection, RhCMV vectors maintained sustained T cell responses to all four malaria antigens in the liver post-challenge. The delayed appearance of blood stage parasites is thus likely due to a T cell-mediated inhibition of liver stage parasite development. As such, this vaccine approach can be used to efficiently test new T cell antigens, improve current vaccines targeting the liver stage and complement vaccines targeting erythrocytic antigens.

## Introduction

Malaria is a global burden and the development of a vaccine is one of the highest priorities for global health research [1, 2]. Malaria is caused by *Plasmodium* parasites with *P*. *falciparum* (Pf) showing the highest mortality whereas Pk occurs naturally in monkeys in Southeast Asia, with frequent infections of humans [3]. *Plasmodium* is transmitted by *Anopheles* mosquitoes, which inoculate sporozoites (Spz) that infect the liver where parasites undergo extensive replication resulting in the release into the bloodstream of thousands of merozoites that infect erythrocytes. Since the pre-erythrocytic (PE) phase involves relatively few parasites and lasts only a few days, PE immunity is limited even in individuals regularly exposed to infection [4]. Instead, infected individuals predominantly develop immunity to erythrocytic parasites. However, antigens expressed on merozoites or erythrocytes display antigenic variation [5]. Thus, naturally acquired immunity to malaria is only partial, highly strain-specific, and short-lived [6]. Thus, a successful vaccine needs to be qualitatively and/or quantitatively different from natural immunity [7].

Sterilizing immunity has been achieved by repeatedly inoculating volunteers with radiation-attenuated Spz (RAS), genetically attenuated Spz (GAP) or chemoprophylaxis with live Spz (CPS) [8, 9]. Indeed, live attenuated Spz are currently in clinical development [10, 11]. However, the large-scale production, shipment, cost and delivery of such a vaccine is challenging. Moreover, large numbers of Spz have to be given intravenously to provide protection and at regular intervals to maintain sufficient immunity over time. Nevertheless, Spz immunizations clearly establish that sterilizing immunity against malaria is possible. The goal for subunit-based vaccines is therefore to match the success of Spz immunization but also to maintain protection over time.

Protection by Spz is predominantly mediated by cellular immunity, particularly interferon (IFN)γ producing CD8+ T cells, characterized as effector memory T cells (T_EM_) [9, 10, 12–16]. Whereas central memory T cells (T_CM_) reside in lymphoid organs from where they expand upon antigen exposure, T_EM_ predominantly reside in non-lymphoid tissues, including the liver, enabling them to respond immediately to incoming pathogens [17]. However, all presently used vaccine vectors ultimately elicit T_CM_ and thus are unable to maintain lasting, liver-resident T_EM_ [18]. Since initial pathogen recognition, expansion, effector differentiation and migration of T_CM_ requires at least one week, the short duration of the liver stage likely enables the parasite to escape control by T_CM_-mediated recall responses [19, 20]. Indeed, the short duration of the liver stage is likely an adaptive trait of the parasite to evade cellular immunity during this vulnerable period. Furthermore, all vaccine strategies currently in development against malaria elicit CD8+ T cells recognizing a limited set of immunodominant, “canonical” MHC-I restricted epitopes within a given antigen resulting in rather focused immune responses that might limit their efficacy [21].

A possible approach to overcome the current limitations of malaria vaccines is the use of CMV as a vector platform. Both HCMV and RhCMV maintain life-long T_EM_ that average 10% of the total circulating memory T cell population [22]. Importantly, HCMV-specific T_EM_ do not show signs of exhaustion: i.e. they retain the ability to produce multiple cytokines [17, 23]. Upon insertion of antigens from Simian Immunodeficiency Virus (SIV), RhCMV elicited and indefinitely maintained high frequency T_EM_ against SIV in RM, including the liver [24, 25]. Importantly, multiple recombinant RhCMV vectors expressing individual SIV antigens and given sequentially or simultaneously provided unprecedented protection against highly pathogenic SIVmac239, with ~50% of vaccinated RM completely controlling and then clearing the SIV infection, the first documented immune-mediated clearance of a lentivirus [26]. RhCMV-based vectors expressing six to nine different antigens derived from *Mycobacerium tuberculosis* (TB) further demonstrated the best known protection of RM against intrabronchial challenge to which RM are exquisitely sensitive [27]. Thus, RhCMV-based vectors are a versatile vaccine platform that has shown unprecedented protection against both viral and bacterial pathogens in RM.

Unexpectedly, RhCMV-vectors differed from all other vaccine vectors not only in their ability to induce and maintain T_EM_, but also in their unprecedented capacity to modulate CD8+ T cell priming [28, 29]. RhCMV strain 68–1 lacking the viral tropism factors UL128 and UL130 elicits CD8+ T cells that exclusively recognize peptides in the context of MHC-II or MHC-E. In contrast, UL128/130-intact recombinants exclusively elicit conventional, MHC-Ia-restricted CD8+ T cells. However, these MHC-I restricted CD8+ T cells recognize “non-canonical” epitopes that are sub-dominant in other vector systems since priming of canonical CD8+ T cells is inhibited by the MHC-I targeting viral protein Rh189 (the RhCMV homologue of HCMV US11) [29]. Thus, RhCMV-based vectors can be programmed to elicit CD8+ T cells to non-overlapping peptides with different restriction elements [30].

To evaluate the potential of CMV-based vectors for malaria vaccines in the RhCMV/RM model, we inserted Pk antigens into RhCMV 68–1 in the presence or absence of Rh189. The four antigen panel (PK4) comprises the Pk homologs of circumsporozoite protein (CSP), apical membrane protein-1 (AMA1), sporozoite surface protein-2 (SSP2/TRAP) and the 42kDa C-terminal fragment of the major merozoite surface protein-1 (MSP1c) [31]. The PK4 panel has been previously tested in RM using various vector platforms given as heterologous prime/boost regimen that demonstrated partial, albeit short-lived protection upon Spz challenge [31–34]. We were able to elicit CD4+ and CD8+ T cell responses to each of the four antigens expressed by RhCMV as well as some antibodies to Spz and blood stage parasites. Here we show that immunization with RhCMV/PK4 significantly delayed the appearance of blood stage parasites. We interpret this result as evidence for an inhibition of liver stage development by Pk-specific T cells. Unlike T cell responses elicited by Spz-infection, the T cell immunity elicited by RhCMV-vectors was maintained over time suggesting that CMV-vectored malaria vaccines can improve and complement current vaccine strategies.

## Results

### Construction of RhCMV/PK4 and ΔRh186-9/PK4

**Fig 1:**
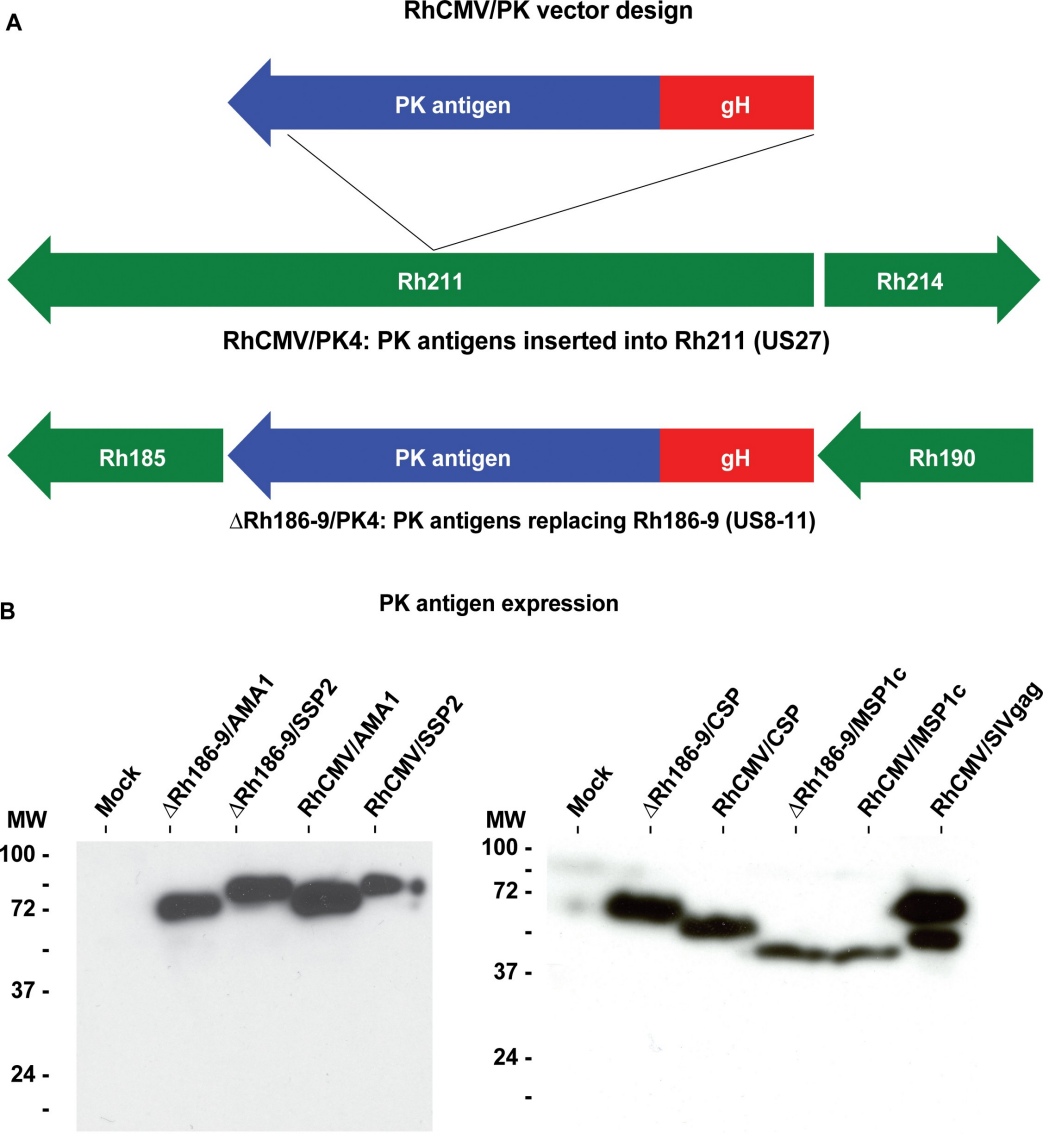
RhCMV vectors expressing Pk antigens. **(**A) Schematic of the Pk antigen expression cassettes inserted into the RhCMV genome. Expression cassettes containing the HCMV gH promoter and codon-optimized synthetic Pk genes encoding for the proteins AMA1, CSP, MSP1c (carboxy-terminal 42 kDa fragment), or SSP2 were inserted into the Rh211 gene (US27 in HCMV) to generate the RhCMV/PK4 panel. To generate the ΔRh186-9/PK4 panel, the expression cassettes were used to replace the gene region Rh186-189 encoding the RhCMV homologs of HCMV US8-11. To facilitate detection, the PK4 antigens were fused to the FLAG epitope sequence at their carboxy-terminus. (B) Immunoblots of Pk antigen expression by RhCMV vectors. Lysates of rhesus fibroblasts infected (MOI = 3) for 24 hours with RhCMV/PK4 or ΔRh186-9/PK4, or RhCMV/SIVgag included as control, were separated by SDS-PAGE and immunoblotted using anti-FLAG antibody. The molecular weight of control proteins is indicated. The left and right panels show the uncropped images of immunoblots of two different gels containing cell lysates that were from the same experiment. The exposure time for each blot was adjusted for optimal detection, with the left panel proteins being detectable after shorter exposure than the proteins shown on the right.

We inserted the four Pk antigens into 68–1 RhCMV and into 68–1 RhCMVΔRh186-189 lacking Rh189 responsible for inhibiting canonical, MHC-I restricted CD8+ T cell priming (deletion of Rh186-188 is irrelevant for T cell modulation [29]). Codon-optimized open reading frames (ORFs) for each of the four antigens were inserted downstream of the HCMV gH promoter [25] with each protein tagged with a FLAG-epitope. We selected the gH promoter since we were unable to recover some of the vectors that expressed the PK antigens under the more commonly used EF1α promoter, presumably since constitutive overexpression of PK antigens is not very well tolerated by infected cells. This is similar to the envelope protein of SIV which also needed to be expressed under the gH promoter to recover recombinant RhCMV [25]. Using bacterial artificial chromosome (BAC) recombineering, each expression cassette was inserted into Rh211 [35] of RhCMV 68–1 to generate the RhCMV/PK4 panel, or the cassette replaced Rh186-189 to generate the ΔRh186-9/PK4 panel (**Fig 1A**). The constructs were validated by next generation sequencing (**S1 Fig**). Upon recovery of virus, expression of the antigens was confirmed by immunoblot (**Fig 1B**). Due to an in-frame internal deletion within the repeat region of CSP in RhCMV/CSP (**S2 Fig**) the molecular weight of the resulting protein is lower compared to ΔRh186-9/CSP (**Fig 1B**). Since this truncation is not expected to reduce the number of potential T cell epitopes we did not repair the deletion.

### Immunogenicity of RhCMV/PK4 and ΔRh186-9/PK4

**Fig 2:**
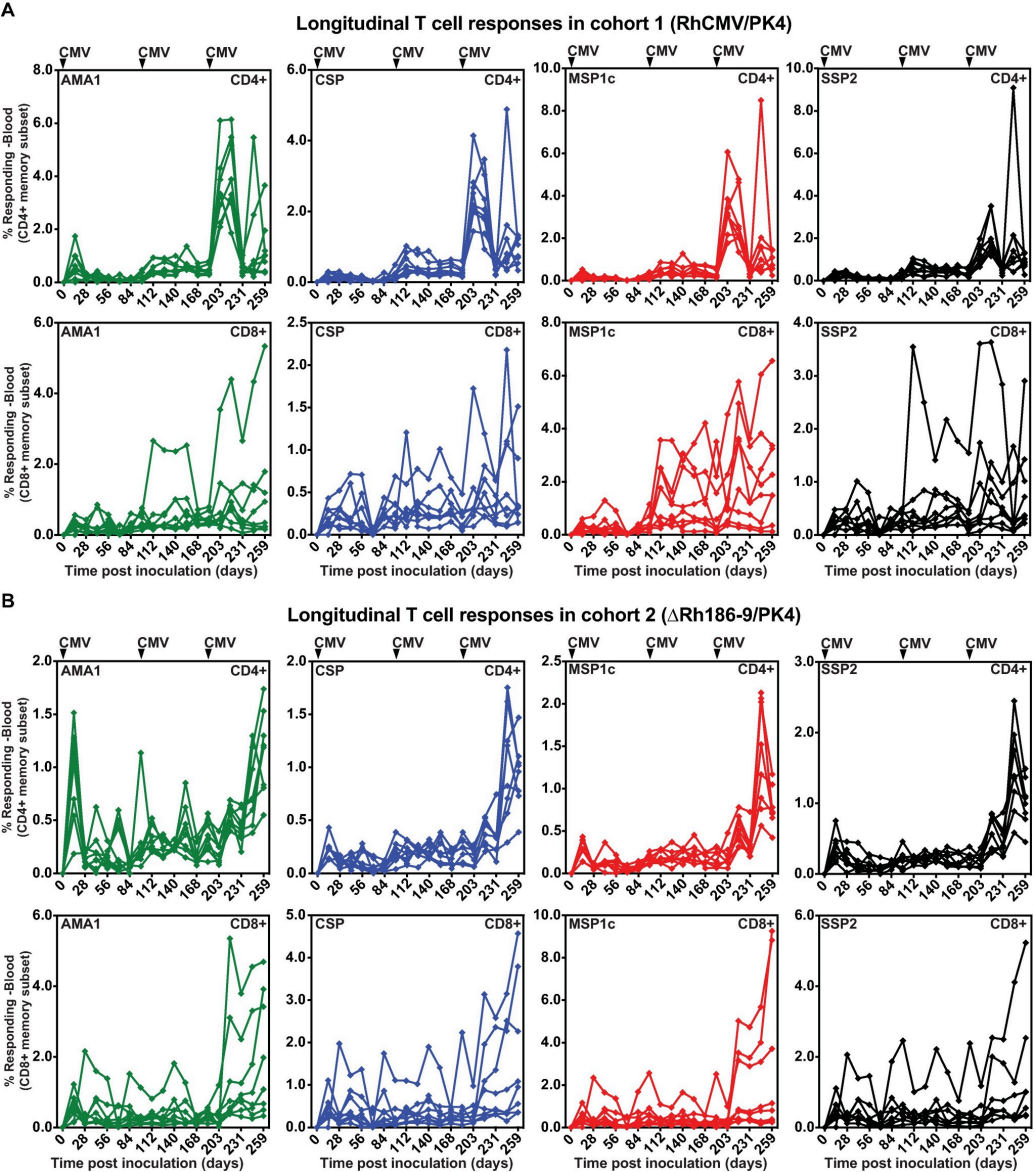
T cell responses to Pk antigens. Frequencies of Pk Ag-specific CD4+ and CD8+ T cells in the blood of animals inoculated with 5x10^6^ PFU of each of the four RhCMV/PK4 recombinants (A) or ΔRh186-9/PK4 recombinants (B) at day 0 and with RhCMV/PK4 on days 98 and 189. The percentage of CD4+ or CD8+ T cells (corrected for memory T cells) responding to each of the four Pk antigens were measured by ICS using overlapping peptide pools at the indicated days. T cell responses are shown for each antigen in each individual animal over time.

RhCMV-based vectors can be used repeatedly and in animals naturally infected with RhCMV due to CD8+ T cell evasion by Rh182 (US2), Rh183 (US3), Rh185 (US6) and Rh189 (US11). Viruses lacking Rh182-189 cannot infect RhCMV-seropositive RM [36] whereas RhCMV lacking Rh186-189, as used here, superinfect such RM [29]. We inoculated 5x10^6^ plaque forming units (PFU) of each of the four RhCMV/PK4 or ΔRh186-9/PK4 vectors subcutaneously (SC) into RhCMV-seropositive male, juvenile RM of cohort 1 (n = 8) or cohort 2 (n = 8), respectively. T cell responses in peripheral blood mononuclear cells (PBMC) were measured by intracellular cytokine staining (ICS) using pools of 15mer peptides, overlapping by 4 amino-acids and spanning each of the PK4 antigens at biweekly intervals. All RM developed CD4+ and CD8+ T cells to all four Pk antigens (**Fig 2**).

**Fig 3:**
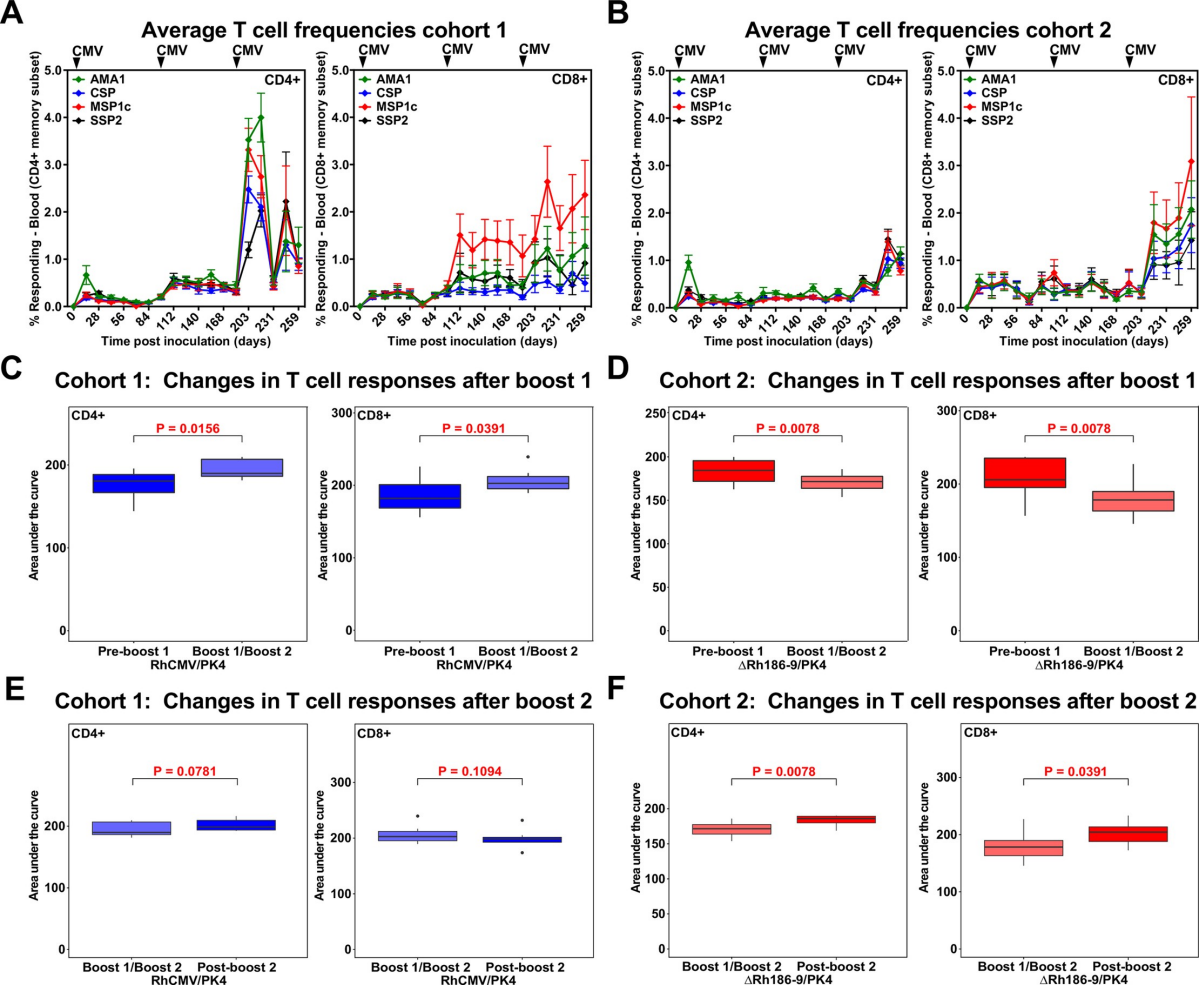
Impact of boosting on T cell responses elicited by RhCMV/PK4 and ΔRh186-9/PK4. **(**A, B) Average frequencies of T cell responses to each of the antigens in cohort 1 (A) or cohort 2 (B) over time. (C-F) Impact of boosting on T cell responses. Statistical analysis of T cell response magnitudes, as determined by measuring the areas under the log10 curve (AUC) of T cell frequencies in each individual RM to all antigens determined by ICS. The boxplots show the median (horizontal line), interquartile range (shaded box), and range (whiskers and outlier points) of the total T cell responses to all antigens. Statistical significance was determined by Wilcoxon test. (C, D) Comparison of the AUC prior to the 1^st^ boost and between boost 1 and 2 within cohort 1 (C) or cohort 2 (D). (E, F) Comparison of the AUC between boost 1 and 2 versus post-boost 2 within cohort 1 (E) or cohort 2 (D) over 91 days between boosts and for 84 days post boost 2.

The immune response in both cohorts was boosted at day 98. We only used RhCMV/PK4 vectors for boosting in both cohorts because we observed in other studies that re-inoculation with Rh189-deleted vectors does not boost T cell responses (data not shown). However, while both CD4+ and CD8+ T cell responses were increased by boosting in cohort 1, average responses did not increase in cohort 2, they even decreased (**Fig 3**). Therefore, we boosted a second time on day 189 which resulted in a significant increase of both CD4+ and CD8+ T cell responses in cohort 2 whereas cohort 1 responses did not increase further (**Fig 3**). After the 2^nd^ boost, CD8+ T cell responses were similar between the two cohorts (**S3 Fig**). However, both total and antigen-specific CD4+ T cell frequencies were significantly higher in cohort 1 compared to cohort 2 (**S3 Fig**). Thus, while all RM displayed robust T cell responses to all antigens after the second boost, it seems that deletion of Rh189 in the first inoculation resulted in reduced induction of CD4+ T cell responses possibly due to viral control by canonical CD8+ T cells. A schematic overview of the prime/boost regimen is shown in **S4 Fig**.

**Fig 4:**
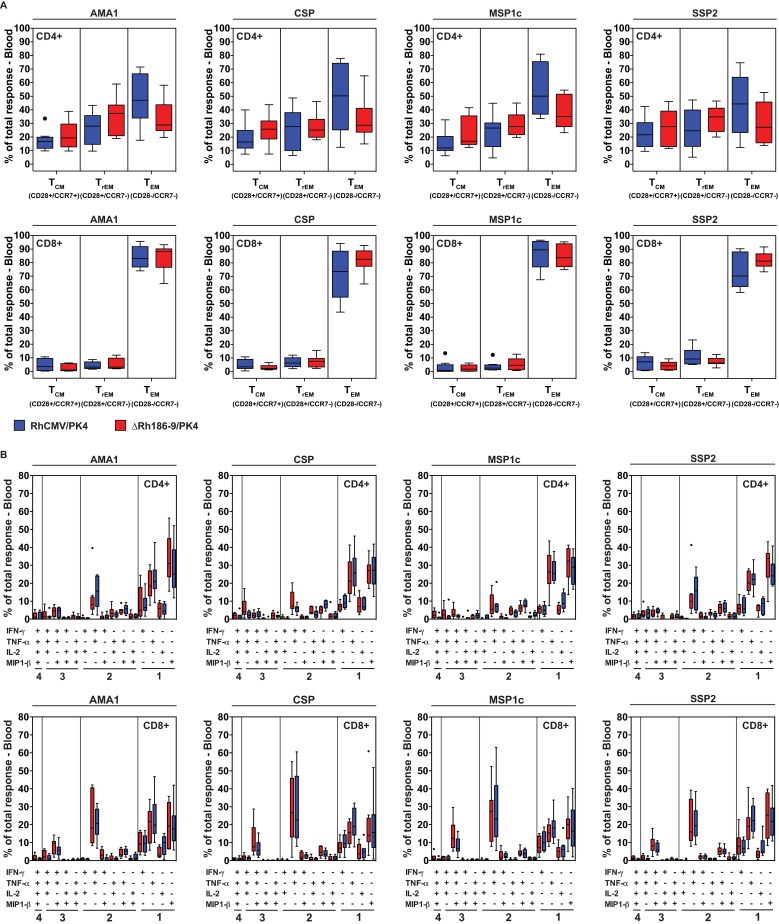
Memory phenotype and cytokine production by Pk-antigen specific T cells elicited by RhCMV. (A) Boxplots comparing the memory differentiation of the RhCMV/PK4 or ΔRh186-9/PK4-elicited CD4^+^ and CD8^+^ memory T cells in peripheral blood responding to each Pk antigen with TNF-α and/or IFN-γ production after the 2^nd^ boost (day 259). Memory differentiation state was based on CD28 vs. CCR7 expression, delineating central memory (T_CM_), transitional effector memory (T_TREM_), and effector memory (T_EM_), as designated. (B) Boxplots comparing the frequency of vaccine-elicited CD4^+^ and CD8^+^ memory T cells in peripheral blood responding to each of the Pk antigens with production of TNF-α, IFN-γ, IL-2 or MIP-1β, alone and in all combinations.

The T cell responses to both HCMV and RhCMV are characterized by their effector memory T cell differentiation and poly-functional phenotype, i.e. the ability to generate multiple cytokines in response to antigen. We therefore analyzed the memory phenotype and individual cytokine production of the T cells elicited to Pk antigens by RhCMV vectors after the 2^nd^ boost. RhCMV vector-elicited PK4-specific CD8+ T cell responses were predominantly effector-differentiated, manifesting an almost exclusive T_EM_ phenotype in both cohorts whereas CD4+ T cell responses displayed a mixed T cell phenotype including central memory, transitional memory and effector memory (**Fig 4A)**. Importantly, a large percentage of malaria antigen-specific CD4^+^ and CD8^+^ T cells produced both TNF-α and IFN-γ (with or without MIP-1β), with the remainder generating individual cytokines including IL-2 (**Fig 4B**). These observations are consistent with previous reports for SIV and TB antigens [25, 27] and highlight the effector functionality of the RhCMV-elicited T cells.

**Fig 5:**
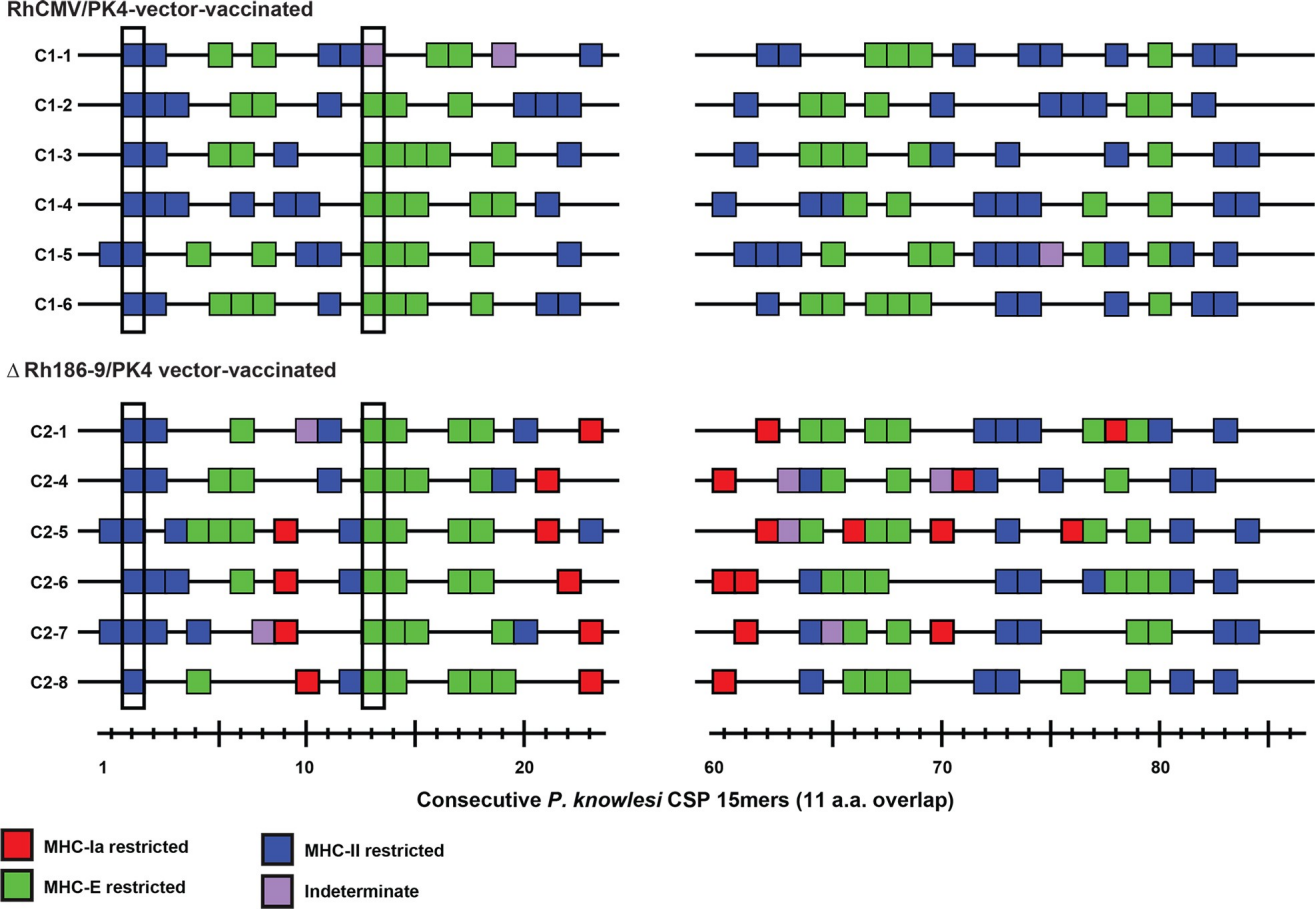
MHC restriction analysis of RhCMV/CSP and ΔRh186-9/CSP-elicited CD8^+^ T cell responses. CSP-specific CD8^+^ T cells were epitope-mapped in six animals of each cohort 1 (upper panel) and 2 (lower panel) using flow cytometric ICS to detect recognition of each consecutive, overlapping 15mer peptide comprising the indicated amino-terminal and carboxy-terminal region of Pk CSP. Peptides resulting in specific CD8^+^ T cell responses are indicated by a box, with the color of the box designating MHC restriction as determined by blocking with the anti-pan-MHC-I mAb W6/32, the MHC-E blocking peptide VL9 and the MHC-II blocking peptide CLIP as previously described [28, 29]. Highlighted are peptides recognized by T cells in every animal.

We previously demonstrated that strain 68–1 RhCMV vectors elicit unconventional CD8^+^ T cell responses that are restricted by MHC-II and MHC-E to SIV and TB antigens [27, 28]. We further showed that 68–1 RhCMV/SIV vectors deleted for Rh186-9 elicit additional CD8^+^ T cells that target “canonical” MHC-Ia restricted epitopes, i.e. epitopes known to be immunodominant in SIV-infected animals, or animals immunized with conventional vector systems [29]. Therefore, it is expected that 68-1-derived RhCMV/PK4 will elicit MHC-II and MHC-E-restricted CD8+ T cells whereas ΔRh186-9/PK4 will additionally elicit MHC-Ia restricted CD8+ T cells. To verify that this is indeed the case we measured the CSP-specific T cell responses of six monkeys in each cohort at the individual peptide level and then determined their MHC-restriction using blocking antibodies (W6/32 for MHC-I) or blocking peptides (invariant chain-derived CLIP for MHC-II and peptide VL9 for MHC-E). We used only the non-repeat regions for this epitope mapping since repeat regions contain the same T cell epitope multiple times. As shown in **Fig 5**, stimulation by CSP peptides of CD8+ T cells from RhCMV/PK4 immunized, cohort 1 animals was either blocked by CLIP or VL9 peptides indicative of MHC-II or MHC-E restriction, respectively. In contrast, stimulation by several CSP peptides recognized by cohort 2 CD8+ T cells was not blocked by either peptide, but was inhibited in the presence of pan-MHC-I antibody W6/32 consistent with classical MHC-I presentation. In addition, we identified two “supertopes”‘, i.e. peptides recognized in each RM, one for each MHC-II and MHC-E (**Fig 5**). As described previously, the ability to present the same peptide regardless of the MHC allotype of each animal is explained by the high conservation of MHC-E and by promiscuous binding to MHC-II [28]. Since these observations are consistent with our previous results obtained with SIV antigens we conclude that the CD8+ T cell response to all four PK antigens was restricted by MHC-II and MHC-E in cohort 1 and by MHC-I, MHC-II and MHC-E in cohort 2.

**Fig 6:**
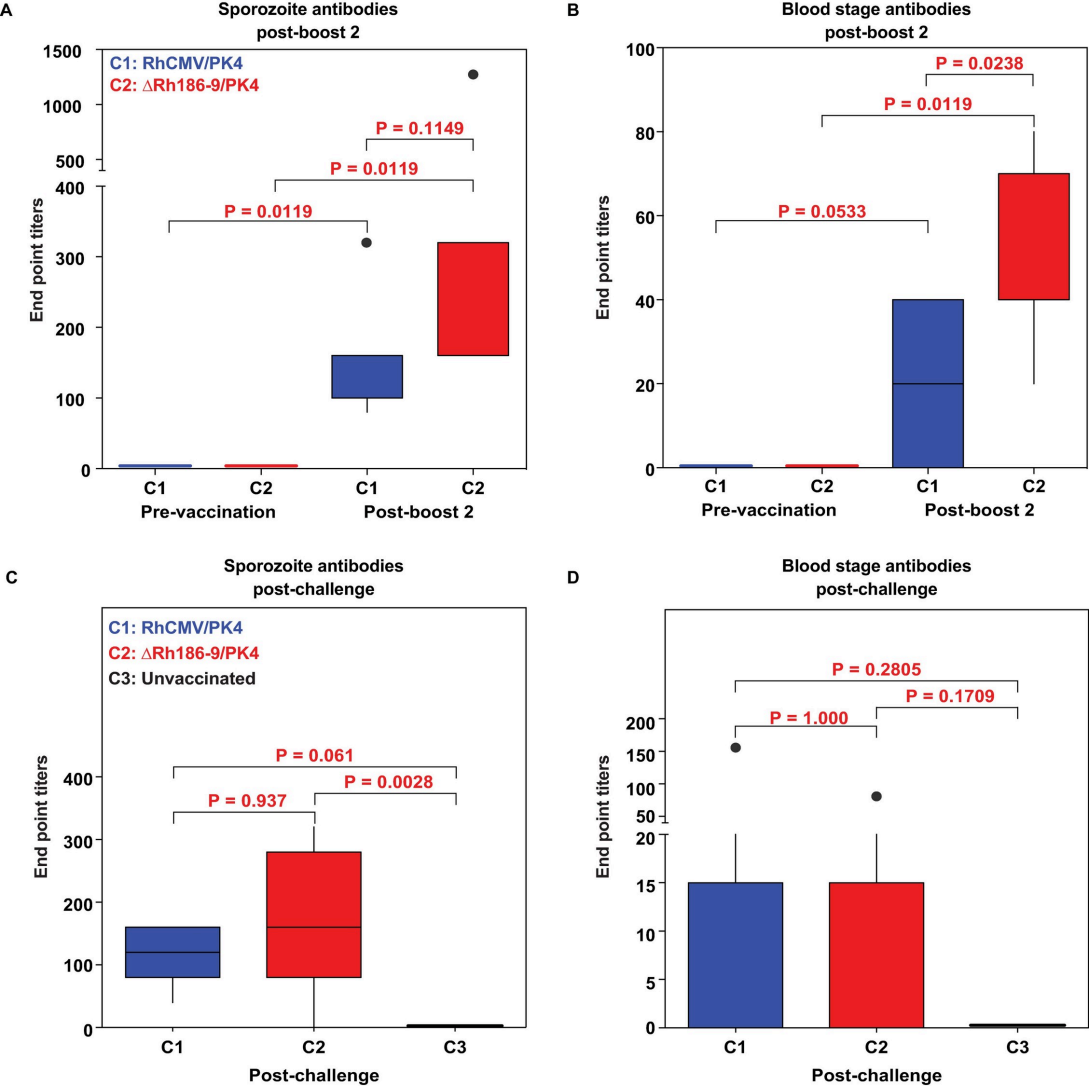
Antibody responses to *P*. *knowlesi* sporozoites and blood stage parasites. **(**A, B) Endpoint titers of IgG antibodies in serum collected from cohort 1 (RhCMV/PK4) or cohort 2 (ΔRh186-9/PK4) prior to immunization on day 0 or on day 203 (post-2^nd^ boost). Pk parasite stage-specific antibodies were measured by IFA to Pk Spz (A) and to Pk-infected RBC (B). (C, D) Endpoint titers to Spz (C) and blood stage parasites (D) determined by IFA at day 14 post-challenge for each animal in the indicated cohorts. Statistical significance for differences in Spz or blood stage antibody titers measured by IFA was determined using the Wilcoxon test. Unadjusted Wilcoxon test p-values comparing IFA results across groups are displayed as boxplots showing the median (horizontal line), interquartile range (box), and range (whiskers and outlier points).

To examine whether the two boost regimen elicited antibody responses to Spz or infected erythrocytes, we used immunofluorescence assays (IFA) of whole parasites to measure antibody titers [37]. In sera collected after the 2^nd^ boost (day 203) we observed modest, but significant, antibody titers to Spz in all immunized animals compared to pre-vaccine sera (**Fig 6A**). Interestingly, the Spz-staining pattern was typical for AMA1 and SSP2/TRAP that localize to intracellular compartments, but lacked typical CSP-staining (data not shown). Antibody responses to blood stage parasites were generally lower than those to Spz (**Fig 6B**) consistent with the fact that only two antigens are expressed in the blood stage (AMA1 and MSP1) whereas three are expressed in Spz (CSP, SSP2, AMA1). Interestingly, cohort 2 animals had higher titers to blood stage parasites than cohort 1 animals, 3 of which were antibody negative, suggesting that deletion of Rh189 has opposite effects on CD4+ T cells and antibody responses.

### Sporozoite challenge

Approximately one month prior to challenge, we assigned an age and sex-matched group of RhCMV-seropositive RM (cohort 3, n = 8) (**S4 Fig**). All cohorts were challenged on the same day with 100 Pk Spz freshly isolated from *Anopheles dirus* mosquitoes that had been fed on Pk-infected RM two weeks earlier[38]. Protection was assessed by daily blood examination starting 6 days after Spz challenge using Giemsa-stained thin blood smears made from ear-prick blood. RM with parasitemias exceeding 2% were treated with chloroquine and artesunate.

**Fig 7:**
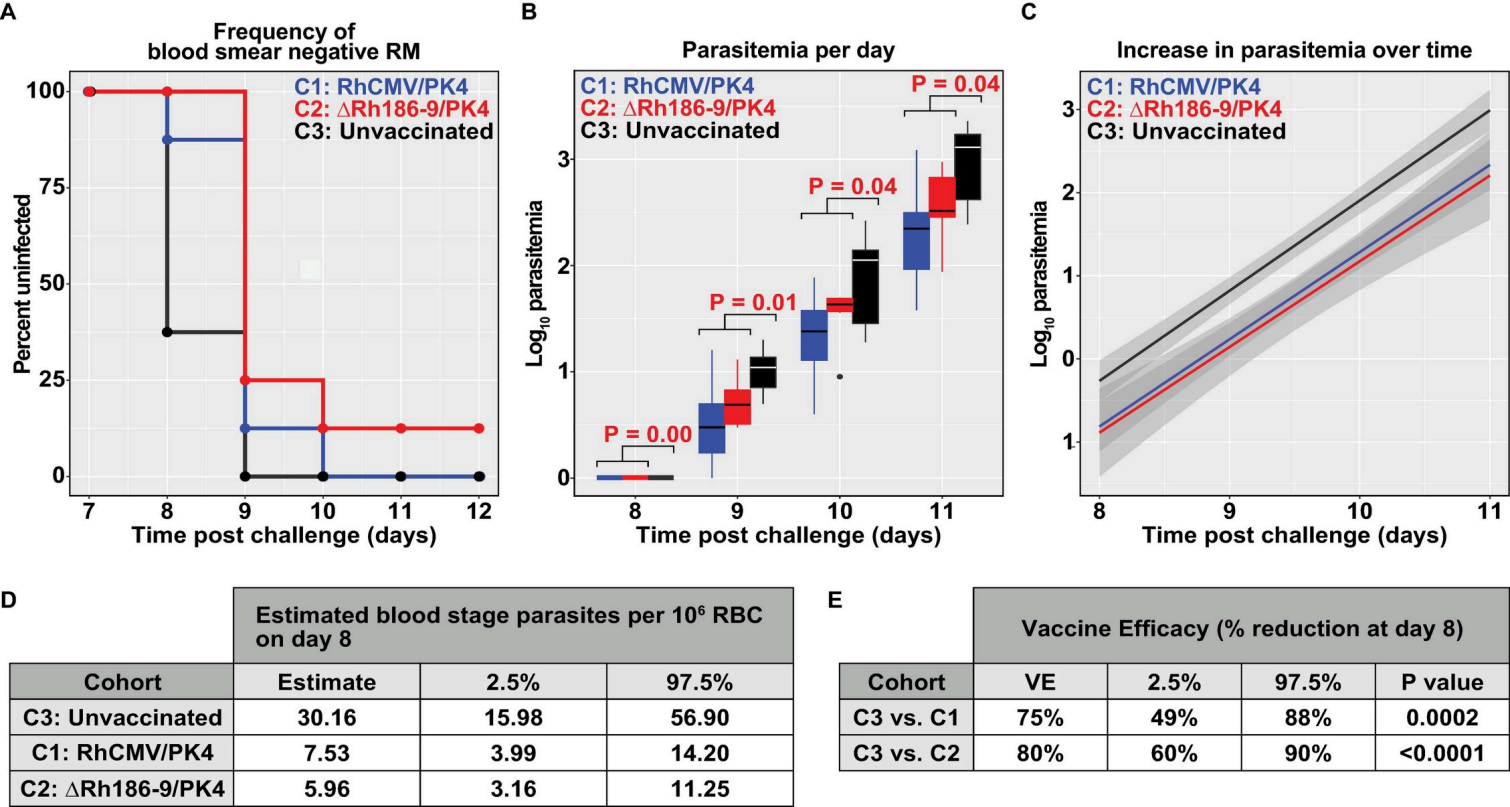
Blood stage parasitemia upon Spz challenge. Each cohort was challenged with 100 Spz at day 0 and blood stage parasitemia was monitored daily by Giemsa stained thin blood smears starting at day 6 post-challenge. (A) Percent of animals without detectable parasitemia at the indicated days post-challenge. The percentage of uninfected RM is shown for each cohort: C1 = animals immunized with the RhCMV/PK4 vector panel, C2 = animals immunized with the ΔRh186-9/PK4 panel, C3 = non-immunized animals. (B) Boxplots of log10 parasitemia per 20,000 RBC at the indicated days show the median (horizontal line), interquartile range (shaded box), and range (whiskers and outlier points) among RM with detectable parasitemia, by day. Statistical significance as determined by unadjusted Wilcoxon test of C1 and C2 versus group C3 is shown above each plot. (C) Linear model fit to the log10-transformed parasitemia values with shaded bands indicating pointwise 95% confidence interval. The parasitemia data to days 8–11 fit a linear regression model and the intercepts for each of the vaccine groups differ significantly (P<0.0001, ANOVA F test, see Methods) from the control group whereas the slopes did not differ (P = 0.9262). This corresponds to an impact on the number of parasites present on day 8. (D) Estimated mean day 8 parasitemia by group and 95% confidence interval are shown in units of 10^6^ RBC. These are estimated from the coefficients of the group terms in a simple linear model relating parasitemia over days 8–11 post-challenge to day and group, as described in Methods. (E) Estimated percent reduction in mean day 8 parasitemia by treated group when compared to control RM, with 95% confidence intervals. These are estimated from the coefficients of the group terms in a simple linear model relating parasitemia over days 8–11 post-challenge to day and group, as described in Methods.

5/8 (62.5%) of control RM were positive for blood stage parasites on day 8 after challenge whereas 15/16 (93.75%) of vaccinated RM remained blood-stage free until day 9 with 3/16 (18.75%) being blood-stage free until day 10 (**Fig 7A, S5 Fig**). One of the RM in cohort 2 remained parasite-free throughout. 75% of control RM, but only 37.5% of cohort 1 and 2 animals, needed to be treated on day 11. On each of the days 8–11 the mean parasitemia was significantly lower in immunized RM compared to control animals (**Fig 7B**). There was no significant difference between the two vaccine groups although its noteworthy that the only sterilely protected RM was in cohort 2. Consistent with T_EM_ being ineffective against blood-stage parasites and the lack of substantial antibodies to the blood stage, the increase in parasitemia over time (as indicated by the slope of the curve) was not reduced in vaccinated RM (**Fig 7C**). Using the parallel increases in parasitemia observed in control and vaccine groups we were able to estimate the average blood stage parasite burden on day 8, the first day parasite were detectable in blood. Control RM were predicted to have 30 parasites per 10^6^ red blood cells (RBC), whereas RhCMV/PK4 and ΔRh186-9/PK4 immunized RM were predicted to have 7.5 and 6 parasites per 10^6^ RBC, respectively (**Fig 7D**). This result corresponds to a 75 and 80% reduction in parasite load in the blood (**Fig 7E**). The most likely explanation for this significant reduction of blood stage parasites is that RhCMV-vector-induced immune responses reduced the liver stage parasite burden, presumably by partially eliminating infected hepatocytes or by inhibiting parasite development in the liver, thus resulting in reduced or delayed release of merozoites. However, once released into the blood stream, the RhCMV-based immune responses were unable to slow parasite growth.

### Post-challenge analysis

**Fig 8:**
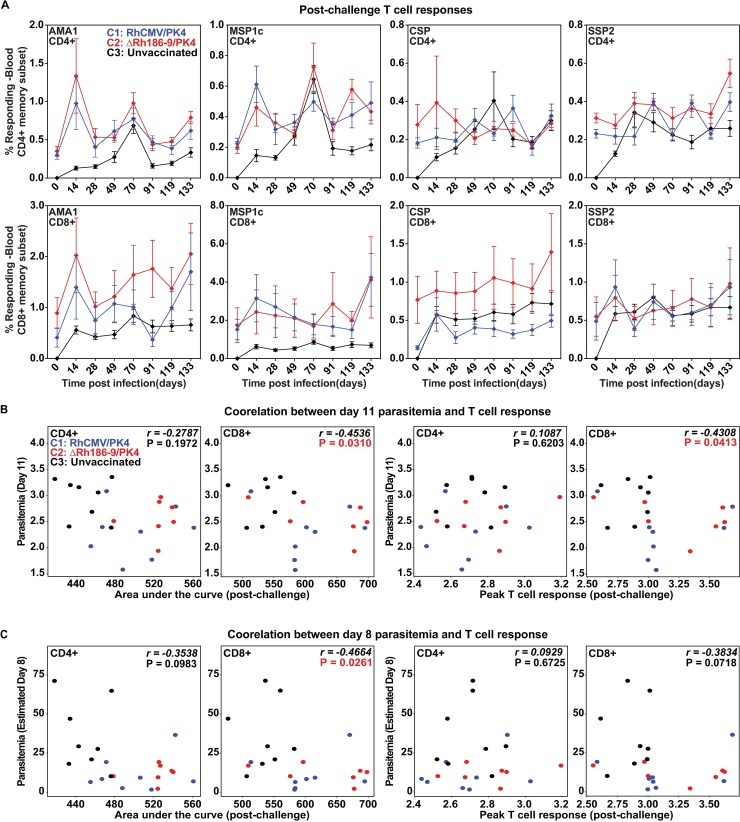
Immune responses in response to parasite challenge. Animals in cohorts 1 and 2 were challenged with 100 PK Spz on day 273 after the first inoculation with RhCMV vectors. Animals in cohort 3 were challenged on the same day. AA) Average CD8+ and CD4+ T cell response frequencies (+/- SD) for each of the four antigens measured in the PBMC of the indicated cohorts by ICS at the indicated time points post-challenge. (B) Post-challenge CD8+ T cell levels correlate with reduced viremia. Scatterplots show association between post-challenge area under the ICS response measurement curve (AUC, left) or the Peak T cell response (right) versus observed Day 11 parasitemia (B) or estimated Day 8 parasitemia (C). Spearman rho (r) values and corresponding p-values shown on each panel indicate significant inverse correlations between CD8+ T cell responses and parasitemia outcome.

All RM in the control group developed *de novo* T cell responses to all four antigens beginning at day 14 post-challenge (**Fig 8A**). However, there was no difference in the AUC prior to challenge and post-challenge in each vaccine group and post-challenge T cell responses did not display a robust increase after challenge (**Fig 8A**). This observation is consistent with the fact that RhCMV-induced T_EM_ do not mount an anamnestic response to antigen [17]. Moreover, epitopes recognized by CD8+ T cells induced by strain 68–1 are not expected to overlap with epitopes recognized by CD8+ T cells induced by Pk, given their unconventional MHC restriction (**Fig 5**). While there might be some overlap between T cells elicited by Spz challenge and canonical, MHC-I restricted CD8+ T cells elicited by Rh189-deleted vectors, only few epitopes would be expected to be shared which is unlikely to affect global T cell response measurements.

We also examined whether Pk-infection boosted antibody responses to Spz or infected erythrocytes using IFA to measure antibody levels at day 14 post-boost. However, we did not observe an increase in antibody responses elicited by RhCMV-vectors (**Fig 6C and 6D**) compared to pre-challenge levels (**Fig 6A**). In fact, the vaccine-induced antibody levels were lower after challenge. One possible explanation is that the antibodies elicited by the vaccine target different epitopes than those elicited by infection. However, Spz-challenge did not elicit a measurable *de novo* antibody response to whole parasites in any of the control RM (**Fig 6C and 6D**). Thus, the transient infection that occurred in all but one RM did not seem to be sufficiently immunogenic to elicit Pk-specific antibodies or boost vaccine-induced antibodies.

In the vaccinated RM that developed blood stage parasitemia there was heterogeneity with respect to levels of parasitemia or day of treatment (**S5 Fig**). Therefore, we examined whether pre-challenge T cell response magnitudes in cohorts 1 and 2 would correlate with the level of parasitemia measured on day 11 or the estimated parasitemia on day 8 (the sterile animal was not included in this analysis). However, neither AUC nor peak levels of CD8+ or CD4+ T cell frequencies post-2^nd^ boost or across the entire vaccination period displayed a significant correlation with parasitemia upon Spz challenge. In contrast, there was a significant inverse correlation between the magnitude of the post-challenge CD8+ T cell responses, either AUC or peak of ICS results, and parasitemia on days 8 and 11 (**Fig 8B**). In contrast, CD4+ T cell responses did not show such a correlation. Since the observed correlation is largely driven by the lower T cell response frequencies and increased parasitemia in the control cohort compared to the vaccine cohorts, it may simply reflect the fact that the vaccinated animals (all of whom had pre-challenge Pk-specific CD8+ T cell responses) were partially protected. However, it is interesting that this correlation was only observed for CD8+ T cells, but not for CD4+ T cells although both T cell sub-populations were strongly induced by vaccination. This result is thus consistent with an important role of CD8+ T cells in delaying blood stage parasitemia.

**Fig 9:**
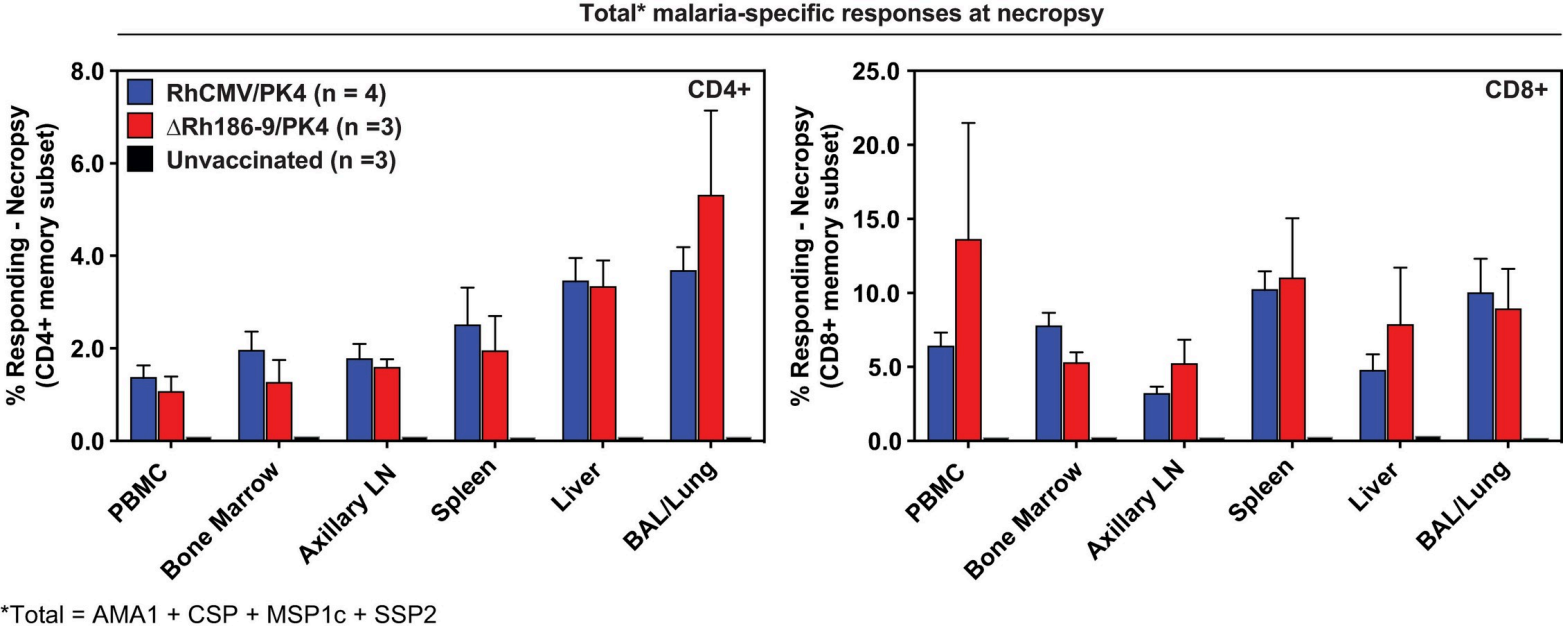
Post-challenge analysis of combined PK4-specific CD4^+^ and CD8^+^ T cell responses in individual tissues. Flow cytometric ICS results of peripheral blood and tissue CD94^+^ and CD8^+^ T cell responses to the peptide mixes comprising each of the four PK antigens in 4 animals of cohort 1 (RhCMV/PK4), three animals of cohort 2 (ΔRh186-9/PK4) and three animals of control cohort 3. The sum of average response frequencies (+SEM), corrected for memory T cells, is shown for the indicated tissues. T cell response frequencies in the control cohort were below the detection limit.

RhCMV-elicited T cell responses are generally maintained in extralymphoid tissues for the life of immunized animals. We therefore determined the tissue distribution of PK4-specific T cells in several RhCMV/PK4 and ΔRh186-9/PK4-immunized animals as well as control animals more than one year post-challenge (**S4 Fig**). Four animals from cohort 1 and three animals from cohorts 2 and 3 were necropsied and T cells were isolated from multiple tissues and analyzed by ICS for each individual antigen (**Fig 9 and S6 Fig**). As we have observed in the past for SIV antigens [24], average CD4+ and CD8+ T cell frequencies ranged from 0.5 to 5% of memory T cells in each tissue with significant T cell responses in the liver against each of the four antigens. Importantly, T cell responses to any of the antigens were undetectable in any tissue of the control animals (**Fig 9**), despite the fact that all control animals had PK4-specific T cells in the blood immediately after Spz challenge (**Fig 8**). Thus, T cell responses elicited by the transient Pk-infection occurring in control animals seemed short-lived. The robust T cell responses measured in the vaccinated cohorts more than one year after challenge are thus due to continued stimulation by RhCMV vectors and not due to the transient Pk-infection that occurred during challenge. These results further support the notion that CMV-vectored malaria vaccines are unique in their ability to maintain high frequency effector memory T cells in the liver.

## Discussion

Our results suggest a significant T cell-mediated control of PE, but not blood stage, parasites by RhCMV-vectored malaria vaccines consistent with the pattern of control expected for a T_EM_-dominated immune response. Although we detected antibody responses after 2 boosts, these were modest and particularly low towards blood stage parasites. Similarly, antibodies to SIV and TB antigens were absent or barely detectable in previous RhCMV-vector studies [25] [27].Since infected erythrocytes do not present antigen and cannot be targeted by T cells, it is not unexpected that RhCMV-mediated immunity was unable to control erythrocytic parasites. The fact that blood stage parasites replicated with similar kinetics in vaccinated and control animals suggests strongly that anti-blood stage antibodies did not impact parasitemia. In contrast, in several previous heterologous prime/boost studies using the PK4 panel, parasites were cleared from the blood in some immunized monkeys, even after reaching significant parasitemia possibly due to the fact that heterologous prime/boost vaccines elicit both T cells and antibodies [32–34]. However, protection by heterologous prime/boost PK4 vaccines was short-lived since protection was lost after 6 months [32]. Although we did not re-challenge the RM in our study, we would expect that the observed delay of blood stage parasitemia will be maintained for life since RhCMV-elicited T_EM_ frequencies do not decrease over time [24]. Indeed, robust blood and tissue T cell frequencies were measured more than one year after challenge in vaccinated animals whereas the T cell response in control animals that were infected during challenge was no longer detectable. Thus, the mechanism of protection by Spz or heterologous prime/boost vaccination and RhCMV-vaccination is likely quite different, as reported in the SIV challenge models [24].

We calculated that between 75–80% fewer parasites were released into the bloodstream in vaccinated as compared to unimmunized animals and one of the immunized animals did not develop parasitemia. This relatively low frequency of sterile protection is similar to most previous Pk challenge studies in RM vaccinated with all or some of the PK4 antigens [31–34]. Only one study reported sterile, albeit short-lived, protection of more than one or two animals per cohort, but this experiment has not yet been repeated [32]. Partial protection was also reported for RAS immunization which protected 5 out of 9 animals upon Spz challenge [39] whereas CPS immunization of 8 RM resulted in sterile protection of 2 RM and delayed parasitemia in 2 RM upon challenge by mosquito bite [40]. In contrast, >90% sterile protection is routinely observed in humans vaccinated with RAS [41–45] or CPS [9, 13, 46, 47] and challenged with *P*. *falciparum* Spz. Thus, Pk challenge of RM seems to be more stringent than Pf challenge of humans. Conceivably, HCMV-based vectors expressing Pf antigens might provide better protection in human challenge studies than in the RhCMV/Pk model. Live-attenuated HCMV-based vaccines are currently in development for HIV and TB, and similar approaches could be used for Pf.

Protection by attenuated Spz is mediated by CD8+ T cells in RM [39] as well as in humans [9, 10]. It is also likely that the delay of blood stage parasitemia by the RhCMV-based malaria vaccines described here was mediated by CD8+ T cells since post-challenge CD8+ T cell responses, but not CD4+ T cells, correlated with decreased parasitemia. Moreover, the increased frequency of CD4+ T cells observed in cohort 1 compared to cohort 2 did not result in increased protection. In fact, the only sterile animal was observed in cohort 2. Thus, our results are consistent with a CD8+-mediated immune mechanism of protection.

We recently described that RhCMV strain 68-1-derived vectors elicit unconventional CD8+ T cell responses exclusively restricted by MHC-II and MHC-E instead of MHC-I [29, 48]. These unconventional CD8+ T cells are an intrinsic feature of the 68–1 strain resulting in MHC-II and MHC-E-restricted responses to heterologous antigens inserted into RhCMV, as previously shown for SIV and TB, as well as to RhCMV proteins [27–29]. We confirmed this unconventional MHC-restriction for malaria antigens by mapping the CSP-specific CD8+ T cells response to individual peptides and demonstrated their MHC-restriction profile with blocking reagents. Consistent with our previous observations, we observed that RhCMV/PK4 elicited CD8+ T cells were exclusively restricted by MHC-II and MHC-E. Thus, the specificities of the CD8+ T cells elicited by the vectors used in cohort 1 are unlike those elicited by Spz-immunization or by other vaccine strategies. The additional deletion of Rh189 in cohort 2 vectors further added MHC-I restricted CD8+ T cells consistent with our previous observation that 68–1ΔRh189 vectors elicit a combination of MHC-I, MHC-II and MHC-E responses to SIV antigens [29, 48]. However, the total T cell response frequency, particularly CD8+ T cells, elicited by ΔRh186-9/PK4 was rather low and required two boosts with RhCMV/PK4 which will only boost unconventional CD8+ T cells. Moreover, the similar delay in parasitemia observed in RM in cohort 1 and cohort 2 suggests that the unconventionally restricted CD8+ T cells are largely, if not completely, responsible for any CD8+ T cell-mediated efficacy. Nevertheless, it is noteworthy that the only sterilely protected RM was in cohort 2, suggesting the possibility that including MHC-I-restricted CD8+ T cells might enhance efficacy in some RM. However, it should be noted that deletion of Rh189 only provides a selected subset of MHC-I epitopes that were termed “canonical” since they are immunodominant in non-CMV contexts [29]. This is reflected by the fact that, on average, only about half the number of peptides was restricted by MHC-I compared to MHC-II or MHC-E in cohort 2 animals. In contrast, wildtype-like vectors in which the RhCMV homologs of UL128 and UL130 were “repaired” elicit much broader CD8+ T cell responses to “non-canonical” MHC-I epitopes that are subdominant in other vector systems [29]. It will thus be interesting to include such repaired vectors expressing Pk antigens in future studies. Since very little is known about the ability of hepatocytes to present malaria antigens in the context of different MHC molecules, immunization with different vector backbones will reveal protective CD8+ T cell sub-populations targeting either conventional or unconventional epitopes.

The four antigens selected in this study are the Pk homologs of Pf antigens that have been extensively studied in various clinical trials using a variety of vaccine platforms. CSP is the antigen in the most advanced malaria vaccine, RTS,S, which partially protects young children from disease [49, 50] and SSP2/TRAP has shown great promise in a number of human challenge studies [19, 48, 51, 52]. Similar to our findings, the blood stage antigens MSP1 and AMA1 expressed by viral vectors did not reduce blood stage parasite growth rates but delayed time to diagnosis suggesting T cell mediated control of the PE stage [48]. Unfortunately, protection by subunit vaccines has not yet achieved the level of protection obtained with attenuated Spz in human challenge studies [53] possibly because the parasite antigens targeted by protective CD8+ T cells elicited by Spz-vaccination are not known. However, various strategies are currently being employed to identify such antigens [54, 55] and these approaches are likely to yield novel vaccine candidates that could be tested in the RhCMV/PK model. A unique aspect of RhCMV-vectors is that multiple vectors can be combined to elicit immune responses to several antigens without interference. In previous work we co-inoculated five vectors expressing five different SIV antigens [24] or four vectors expressing nine different *M*. *tuberculosis* antigens [27]. In each case, we were able to elicit T cell responses to each of the antigens without observing any interference from the others. In contrast, the immune responses to MSP1 dominated over AMA1 and SSP2/TRAP in humans inoculated with pox- and adenoviral vectors [48]. Thus, the RhCMV/PK system might be an ideal platform to rapidly test large numbers of potential vaccine candidates for protection.

The tissue distribution of T cells is an inherent feature of RhCMV-vectored vaccines that is irrespective of the heterologous antigen used or the route of inoculation. As observed in previous studies with SIV antigens [24], CD8+ T cell responses to each of the four PK antigens were particularly elevated in the liver, a result that is consistent with liver-localized CD8+ T cells controlling parasites in the liver. The unique ability of RhCMV vectors to elicit and indefinitely maintain a robust and sustained T cell response to malaria antigens in the liver renders vectors based on HCMV attractive for further development of vaccines targeting the liver stage either by themselves or in combination with traditional vaccines or vaccine vectors. Since the vast majority of individuals living in malaria-endemic regions are naturally infected with HCMV, any malaria vaccine must already be immunogenic in the context of a HCMV-immune host environment. Therefore, HCMV-vectored malaria vaccines can be combined with any other malaria vaccine to either broaden the T cell responses and epitope targeting to a given antigen, or to include a potent T cell component to an antibody-inducing vaccine, or additional antigens into existing vaccines.

## Conclusion

This is the first report demonstrating control of malaria by unconventional T_EM_-inducing RhCMV vectors. The delay in the appearance of blood stage parasites suggests that these T cells significantly controlled the liver stage of the parasite. As such, this study established proof of principle for this novel approach for malaria vaccine development. There are multiple possibilities to further improve protection including, but not limited to, programming different CD8+ T cells using genetically distinct HCMV vectors, screening multiple novel antigens, and combining HCMV vectors with other vaccine strategies.

## Materials and methods

### Construction of recombinant RhCMV

FLAG-epitope tagged, codon-optimized Pk genes were synthesized by GeneArt based on Pk strain H: AMA1 (XP_002259339), CSP (XP_002259002), the C-terminal 328 AA of MSP1 (XP_002258582) and SSP2/TRAP (XP_002259987). Using the RhCMV 68–1 BAC [56] we generated the four vector panel RhCMV/PK4 by inserting expression cassettes of ORFs under control of the HCMV gH promoter into Rh211 using homologous recombination [25]. The ΔRh186-9/PK4 panel was generated by replacing the genomic region NT196625-NT199855 with the gH/PK expression cassette in the 68–1 BAC [29]. All BACs were analyzed by restriction digest and by NGS on an Illumina MiSeq sequencer to confirm genomic integrity. BACs were electroporated into rhesus fibroblasts to reconstitute virus. Pk antigen expression was confirmed by immunoblot of infected cell lysates and vaccine stocks were generated by the OHSU Molecular Virology Support Core.

### Ethics statement

Purpose-bred adult rhesus macaques (*Macaca mulatta)* of Indian origin were used either at the Walter Reed Army Institute of Research/Naval Medical Research Center (WRAIR/NMRC) or the Oregon National Primate Research Center (ONPRC). Both facilities are accredited by the Association for Assessment and Accreditation of Laboratory Animal Care. The experiments were conducted in compliance with the Animal Welfare Act in accordance with the “Guide for the Care and Use of Laboratory Animals,” Institute of Laboratory Animals Resources, National Research Council and approved by the respective Institutional Animal Care and Use Committees (IACUC) that adhere to national guidelines established in the Animal Welfare Act (7 U.S.C. Sections 2131–2159) and the Guide for the Care and Use of Laboratory Animals (8th Edition) as mandated by the U.S. Public Health Service Policy. The animal study protocols were reviewed and approved either by the IACUC of the WRAIR/NMRC or ONPRC in compliance with all applicable federal regulations governing the protection of animals and research under NMRC protocol 14-IDD-24LS (Production of malaria parasites using rhesus monkeys) or ONPRC protocol IS00002413–0963 (An Effector Memory T Cell-Inducing Subunit Vaccine against Malaria). In this study the major risk to the animals was from the malaria infection. Harm from malaria infection was minimized by treating with anti-malarial drugs at a parasitemia level low enough to prevent serious illness.

### Rhesus macaques

For challenge experiments, Pk Spz were generated at the WRAIR/NMRC in two purpose-bred, male RM of Indian origin from the WRAIR breeding colony at Covance, Texas. RM were splenectomized prior to infection with blood-stage parasites and feeding of mosquitoes. For vaccine studies, 24 purpose-bred, pedigreed, male RM were used at ONPRC. At assignment, these RM were positive for RhCMV but free of Macacine herpesvirus 1, D-type simian retrovirus, simian T-lymphotrophic virus type 1, simian immunodeficiency virus, and TB. The RM were housed in Animal Biosafety Level-2 rooms with insect control. Animals were fed commercial monkey chow (Purina) and fresh fruit and vegetables provided daily; water was provided at all times via automatic watering system. All non-human primates were provided with environmental enrichment in the form of manipulanda (such as toys, wood stick, or mirror, etc.). Additionally, the enrichment program uses toys and food inside and outside the cage to promote species-specific behavior, such as a foraging boards and visualization of conspecifics as well as Music and TV on a rotational basis. Additional environmental enrichment included cage perches, daily interactions with animal care staff, daily treats and fresh fruits and vegetables as a food enrichment.

RM were sedated with ketamine HCl or Telazol for subcutaneous vaccine administration or intravenous Spz administration. All animals with malaria infections were closely monitored and treated with anti-malarial drugs, and adjunctive therapy as needed.

### Malaria parasites

*Plasmodium knowlesi* H strain parasites were derived from stocks at the NIH.

### Mosquitoes

*Anopheles dirus subspecies A* were provided by the Laboratory of Malaria and Vector Research, NIAID, NIH.

### Immunization

RM of cohorts 1 (n = 8) and 2 (n = 8) were immunized by subcutaneous administration of 5x10^6^ PFU of each RhCMV/PK4 or ΔRh186-9/PK4 vector, respectively, on day 0 by inoculating each of the four vectors in a separate site (right arm, left arm, right leg, left leg). RhCMV/PK4 vectors were administered twice again to both cohorts on days 98 and 190.

### T cell assays

PK-specific CD4+ and CD8+ T cell responses were measured in PBMC by ICS [24, 25, 36]. Briefly, PBMC were incubated with consecutive 15mer peptide mixes (11 amino acid overlap) comprising the Pk proteins and the co-stimulatory molecules CD28 and CD49d (BD Biosciences) for 1h, followed by addition of Brefeldin A (Sigma-Aldrich) for an additional 8hrs. Co-stimulation without peptides served as background control. As previously described [28, 29], the MHC restriction (MHC-Ia, MHC-E, MHC-II) of a peptide-specific response was determined by pre-incubating isolated mononuclear cells for 1 hr at room temperature (prior to adding peptides and incubating per the standard ICS assay) with the following blockers: 1) the pan anti-MHC-I mAb W6/32 (10mg/ml), 2) the MHC-II-blocking CLIP peptide (MHC-II-associated invariant chain, amino acids 89–100; 20μM), and 3) the MHC-E-blocking VL9 peptide (VMAPRTLLL; 20μM). Blocking reagents were not washed, but remained throughout the assay. Stimulated cells were fixed, permeabilized and stained [24, 25, 36] using combinations of the following fluorochrome-conjugated mAbs: SP34-2 (CD3; Pacific Blue, Alexa700), L200 (CD4; AmCyan, BV510), SK-1 (CD8; PerCP-Cy5.5), MAB11 (TNFα; FITC, PE), B27 (IFNγ; APC), FN50 (CD69; PE-TexasRed), B56 (Ki-67; FITC), and in polycytokine analyses, JES6-5H4 (IL2; PE Cy-7). Data was collected on an LSR-II (BD Biosciences). Analysis was performed using FlowJo software (Tree Star). Lymphocytes were gated for CD3+ and progressive gating on CD4+ and CD8+ T cell subsets. Antigen-responding cells in both CD4+ and CD8+ T cell populations were determined by their intracellular expression of CD69 and one or more cytokines. After subtracting background, the raw response frequencies were memory corrected[24, 25, 36] using combinations of the following mAbs to define the memory vs. naïve subsets: SP34-2 (CD3; Alexa700, PerCP-Cy5.5), L200 (CD4; AmCyan), SK-1 (CD8; APC, PerCP-cy-5.5), MAB11 (TNFα; FITC), B27 (IFNγ; APC), FN50 (CD69; PE), CD28.2 (CD28; PE-TexasRed), DX2 (CD95; PE), 15053 (CCR7; Pacific Blue), and B56 (Ki-67; FITC). For memory phenotype and polycytokine analysis of Pk antigen-specific T cells, all cells expressing CD69 plus one or more cytokines were first Boolean gated, and then this overall Ag-responding population was subdivided into the subsets of interest on the basis of surface phenotype or cytokine production pattern [24, 25, 36].

Prior to necropsy, RM were euthanized a with sodium pentobarbital overdose (>50 mg/kg) and exsanguinated via the distal aorta. Mononuclear cell preparations were obtained from blood, bone marrow, lymph nodes, spleen, liver, intestinal mucosa, colon and broncho-alveolar lavage (BAL) as previously described [25].

### Immunofluorescence assay (IFA)

RM sera were tested by IFA for reactivity to Spz and asexual erythrocytic stages of Pk as previously described [37]. To determine IgG titers erial dilutions of the sera were incubated for one hour at 37°C, washed and developed with FITC-labeled goat anti-human IgG for 30 minutes at 37°C in the presence of 0.005% Evan’s blue. End-point IgG titers were determined under a fluorescence microscope as the last titer showing specific reactivity to the parasite stage and recorded as digital pictures.

### Production of Pk*-*infected mosquitoes

One splenectomized RM was anesthetized with ketamine and acepromazine and infected by intravenous injection of cryopreserved red blood cells infected with Pk. Female mosquitoes were used 5–7 days after emerging from pupae. Pint cartons of 100 mosquitoes were starved for 8hrs prior to the feeding; cartons were placed against the shaved skin of the infected RM, anesthetized as above, under drapes for darkness. After feeding for 30 minutes, mosquitoes not engorged with blood were removed, and the remaining mosquitoes were maintained at 26°C and 85% humidity. Cotton pads soaked in sugar solution were changed daily.

### Spz challenge

*Anopheles dirus* mosquitoes were used on day 17 after they had fed on a Pk-infected RM. Spz were dissected by the Ozaki method into M199 medium (Sigma) with 5% normal RM serum. For challenge at day 273 post-inoculation with RhCMV, 100 Pk Spz were injected intravenously in 1 ml RPMI1640 with 5% normal rhesus serum. Starting at day 6 after challenge, blood was obtained by skin prick for thin film slides. After Giemsa staining, blood was examined under ×1000 magnification until 20,000 RBC were examined. Infected animals were treated when parasitemias reached 2% by intramuscular injections of artesunate (5 mg/kg single dose) and chloroquine diphosphate (25 mg/kg for three consecutive days). Animals were monitored for parasitemia the day after treatment and on days 17 and 20 post-challenge to ensure effectiveness of treatment in clearing malaria infections. The sterile animal was not treated but monitored daily until day 23 post-challenge.

### Statistical analysis

Statistical analysis was conducted using R[57]. Data transformations were conducted prior to analysis (log10 transformations were applied to parasitemia and ICS measurements). Statistical significance was evaluated using a 5% type I error threshold on unadjusted values, and a 5% type I family-wise error threshold on adjusted p-values using the Holm method for multiplicity adjustment to control false discoveries across T cell frequencies measured by ICS or endpoint antibody titers measured by IFA[58]. All p-values shown are unadjusted except where noted. Boxplots were created using standard Tukey rules: boxes show interquartile range, IQR, with line at median, and whiskers extend to most extreme datapoints within 1.5*IQR from 25^th^ and 75^th^ percentiles, and outlier values outside of whiskers are plotted as points. P-values are shown across boxes only if significant (p≤0.05).

Comparisons across groups were conducted using two-sided Wilcoxon tests, and comparisons across time points were conducted using paired two-sided Wilcoxon tests[59].

Standard linear regression analysis was applied to relate the log10 parasitemia on days 8 through 11 to the day (coded as days post challenge minus 8, so the intercept is estimated day 8 log10 parasitemia). The model fit was good, especially with a simple model relating log10 parasitemia on each day to a group-specific intercept and a slope (adjusted R^2^ = 0.795), and also with a more complex model that allows the slope to vary by treatment group (adjusted R^2^ = 0.79). We employed standard ANOVA F-tests [59] to evaluate the significance of the interaction term (reflecting varying slope by treatment group) and found that the evidence did not support inclusion of the interaction term (P = 0.93) when comparing these two models, but when comparing the simpler model to the trivial model that ignores treatment group (adjusted R^2^ = 0.75), the ANOVA F-test P-value is highly significant (P = 2.3e^-5^), supporting the inclusion of group-specific intercepts, but not group-specific slopes, in the final model. Estimated Day 8 parasitemia for each RM was determined from the model fit. Due to discretization, observed Day 8 parasitemia values are either 0 or 1, but estimated Day 8 values from the model provide sufficient variation for immune correlates evaluation.

Vaccine efficacy was evaluated as percent reduction in estimated Day 8 parasitemia compared to the control group, with confidence intervals for the difference and p-values given by the linear model fit described above (using the simple model relating log10 parasitemia to days post infection minus 8, with group-specific intercepts), using the T distribution and test as is standard for evaluating coefficients of regression models.

Correlations were evaluated using Spearman’s method [60] (rank-transformation followed by Pearson correlation), and tested using the cor.test (., method = “spearman”) method in R.

## Supporting information

S1 Fig Next Generation sequence analysis of RhCMV vectors containing Pk antigens. The bacterial artificial chromosomes (BACs) of recombinant RhCMV constructs were sequenced by NGS and analyzed. All sequencing reads passing quality control were aligned to the de novo assembled consensus sequence of the viral genome. The consensus sequence was aligned with the parental RhCMV 68–1 BAC (Genbank Accession JQ795930) and the ORF map of the consensus sequences are shown. The bar indicates the percentage of nucleotide identity between the test and the reference sequences with green being 100% identical. The BAC cassette (green ORFs) is flanked by loxP sites (red). The only sequence difference between the parental BAC and the individual constructs is at the site of Pk antigen insertions (blow up below the full genome). In the RhCMV/PK4 vectors the antigens disrupt ORF Rh211. In the ΔRh186-9/PK4 vectors the antigens replace the ORFs Rh186, Rh187, Rh188 and 189.
(PDF)

S2 Fig In frame deletion of CSP repeats encoded by RhCMV. Nucleotide sequence alignment and in silico translation of the CSP insert ΔRh186-9/CSP (upper sequence) and in RhCMV/CSP (lower sequence). The sequence was generated from DNA of virus isolated from the supernatant of infected rhesus fibroblasts. The in-frame deletion in the CSP region of RhCMV/CSP resulted in an internal truncation of the repeat region.
(PDF)

S3 Fig Comparison of T cell responses elicited by RhCMV/PK4 and ΔRh186-9/PK4. **(**A) Comparison of T cell response magnitudes, as determined by measuring the areas under the log10 curve (AUC) of T cell frequencies for each individual RM determined by ICS, between cohort 1 (RhCMV/PK4) and Cohort 2 (ΔRh186-9/PK4) over the entire immunization period. The boxplots graph shows the average (within 95% CI) median (horizontal line), interquartile range (shaded box), and range (whiskers and outlier points) of the total T cell responses to all antigens, whereas the table shows the p-values for the comparisons of each of the antigens individually. Statistical significance was determined by Wilcoxon test and we applied the Holm p-value adjustment method for controlling the family-wise error rate over the four genes. (B) Comparison of the peak T cell response over the immunization phase either for all antigens (boxplot graph) or for each antigen individually (table). Statistical analysis was as in A). (C) Comparisons of T cell response magnitudes (AUC) determined for cohort 1 and cohort 2 after the 2nd boost. Statistical analysis was as in A). (D) Comparisons of peak T cell response magnitudes determined for cohort 1 and cohort 2 after the 2nd boost. Statistical analysis was as in A).
(PDF)

S4 Fig Schematic of animal experiments. Schematic of the RM cohorts, immunization schedule, challenge time points, post-challenge analysis and necropsy. Stars indicate the days when sera were collected for analysis of the antibody response. T cell functional assays indicate the day of blood collection for T cell phenotype analysis. The week (wk) post-vaccination of the animals necropsied in each cohort is indicated.
(PDF)

S5 Fig Number of infected red blood cells per 20,000 cells for each animal at the indicated days post-challenge. Parasitemia was determined as described in the Materials and Methods. Animals were treated with anti-malarial drugs when parasites exceeded 2% parasitemia (>400 infected RBC) on the indicated days.
(PDF)

S6 Fig Post-challenge analysis of individual PK4-specific CD4^+^ and CD8^+^ T cell responses in individual tissues. Flow cytometric ICS results of peripheral blood and tissue CD4^+^ and CD8^+^ T cell responses to the peptide mixes comprising each of the four PK antigens in 4 animals of cohort 1 (RhCMV/PK4), 3 animals of cohort 2 (ΔRh186-9/PK4) and 3 animals of control cohort 3. The average response frequencies (+SEM), corrected for memory T cells, is shown for the indicated tissues for each of the antigens.
(PDF)
