# Supplementary figures and images for: Cytomegalovirus vectors expressing Plasmodium knowlesi antigens induce immune responses that delay parasitemia upon sporozoite challenge
Source: PLoS One. 2019 Jan 23;14(1):e0210252. doi: 10.1371/journal.pone.0210252 (PMC6343944; doi:10.1371/journal.pone.0210252)

RhCMV/PK4 BAC

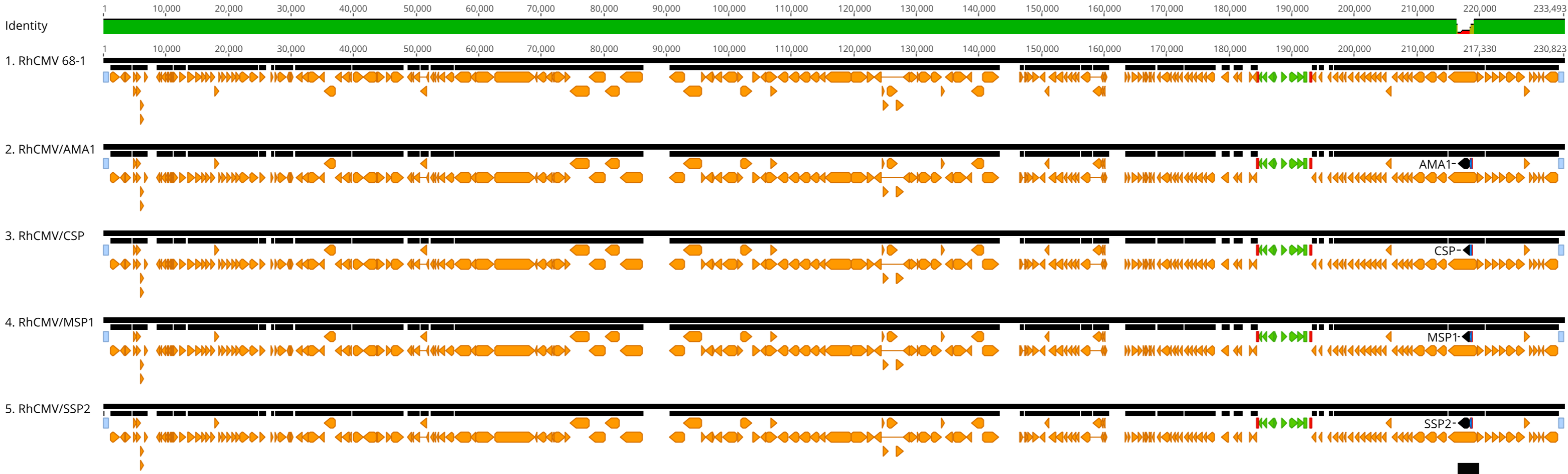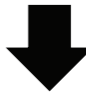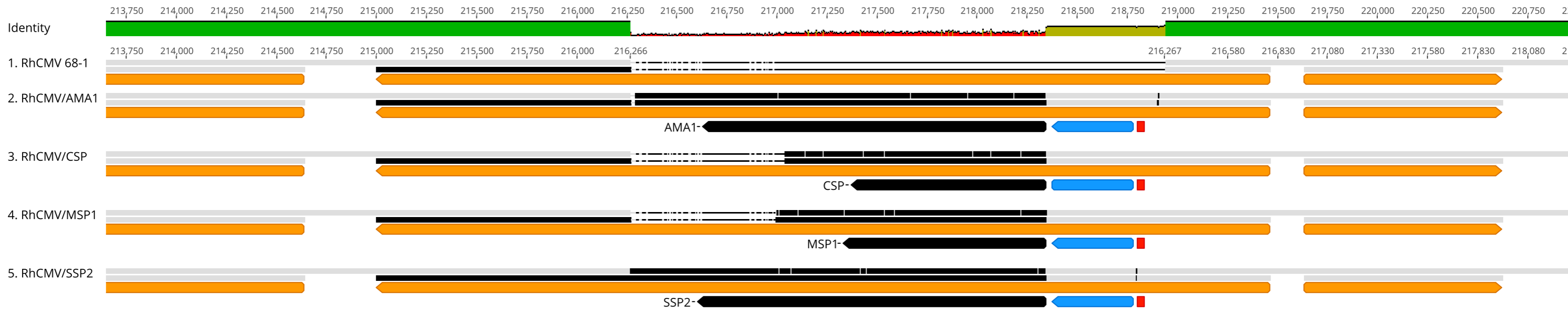

RhCMVΔRh186-9/PK4 BAC

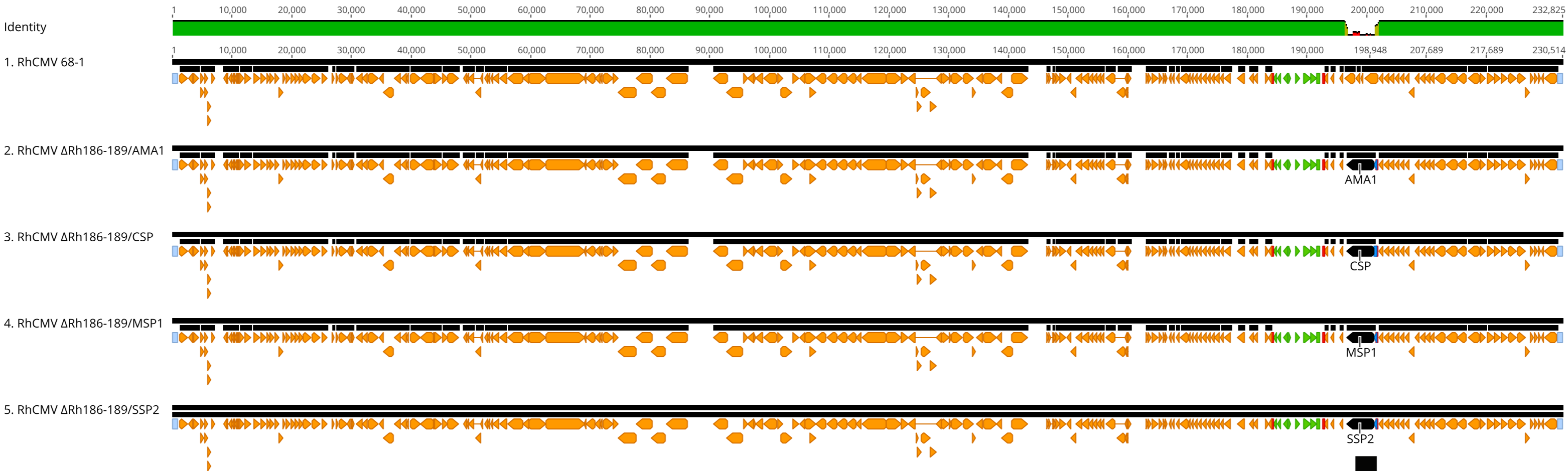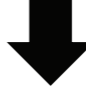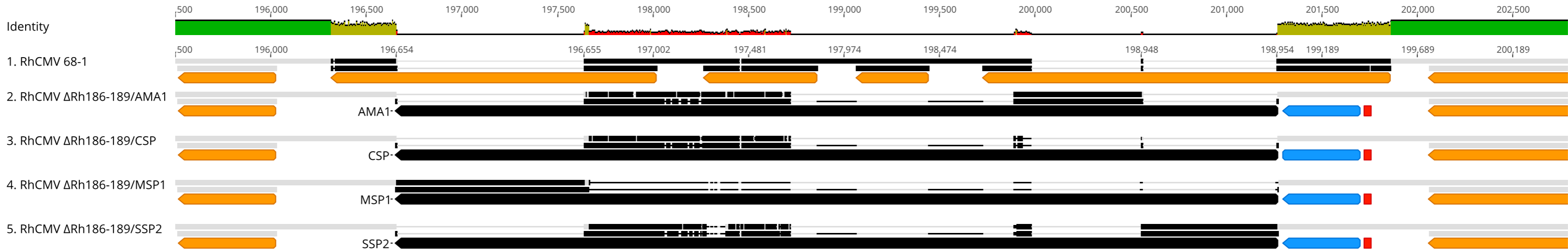

Supplement: S1 Fig — The bacterial artificial chromosomes (BACs) of recombinant RhCMV constructs were sequenced by NGS and analyzed. All sequencing reads passing quality control were aligned to the de novo assembled consensus sequence of the viral genome. The consensus sequence was aligned with the parental RhCMV 68–1 BAC (Genbank Accession JQ795930) and the ORF map of the consensus sequences are shown. The bar indicates the percentage of nucleotide identity between the test and the reference sequences with green being 100% identical. The BAC cassette (green ORFs) is flanked by loxP sites (red). The only sequence difference between the parental BAC and the individual constructs is at the site of Pk antigen insertions (blow up below the full genome). In the RhCMV/PK4 vectors the antigens disrupt ORF Rh211. In the ΔRh186-9/PK4 vectors the antigens replace the ORFs Rh186, Rh187, Rh188 and 189. (PDF) [file pone.0210252.s001.pdf]

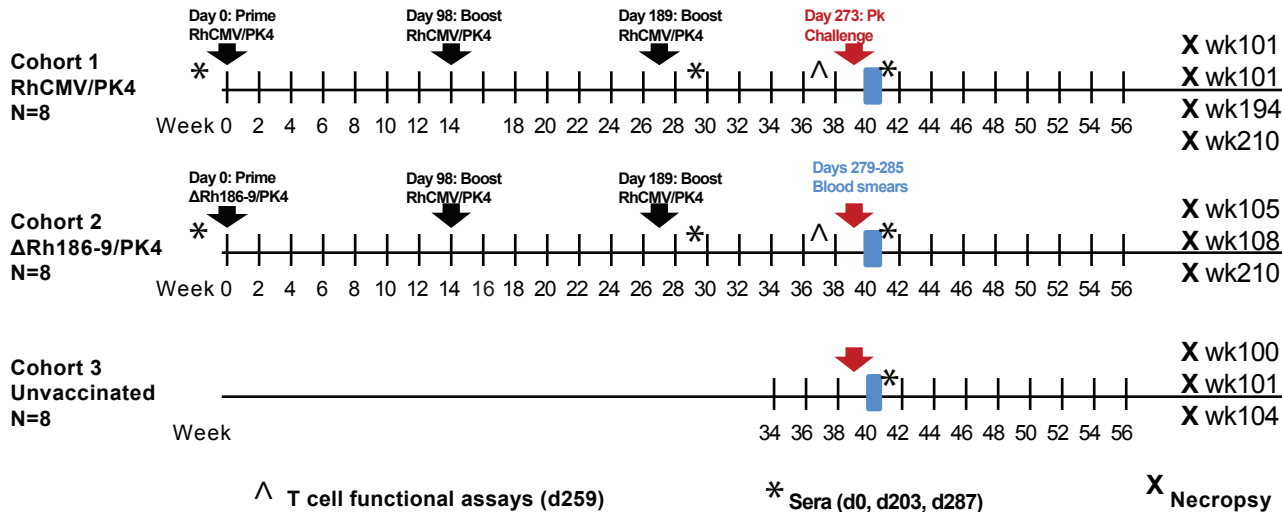

Supplement: S4 Fig — Schematic of the RM cohorts, immunization schedule, challenge time points, post-challenge analysis and necropsy. Stars indicate the days when sera were collected for analysis of the antibody response. T cell functional assays indicate the day of blood collection for T cell phenotype analysis. The week (wk) post-vaccination of the animals necropsied in each cohort is indicated. (PDF) [file pone.0210252.s004.pdf]

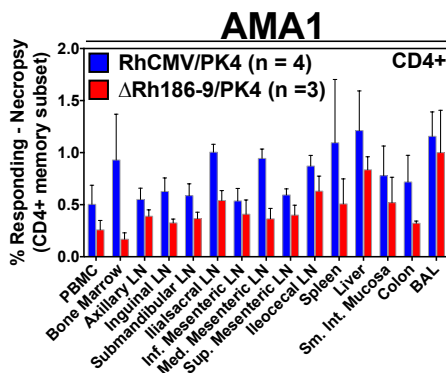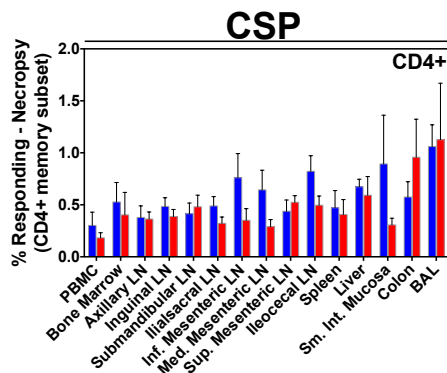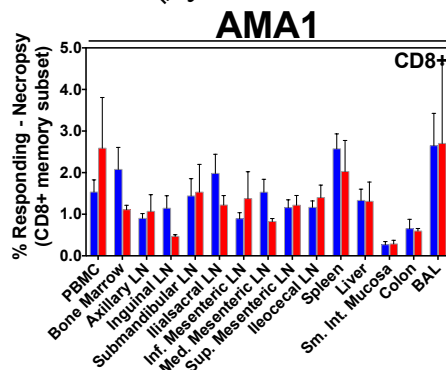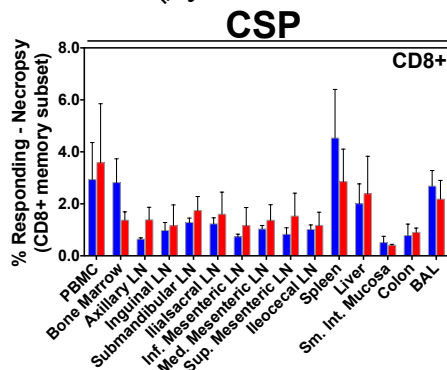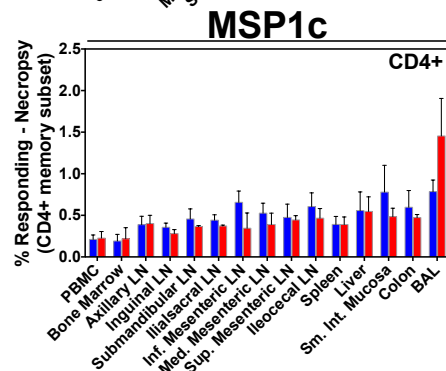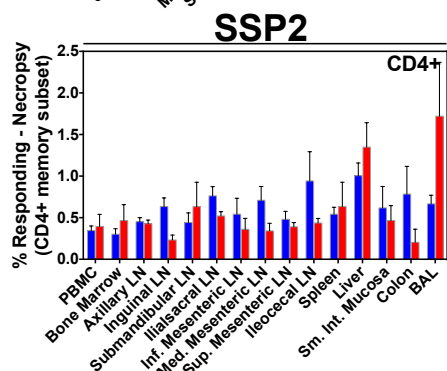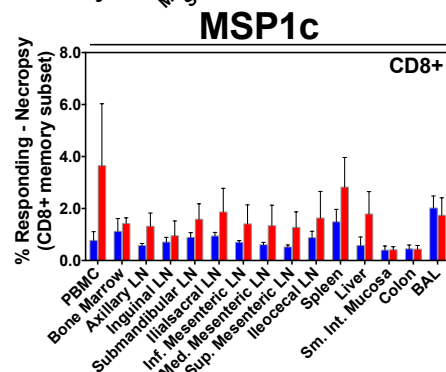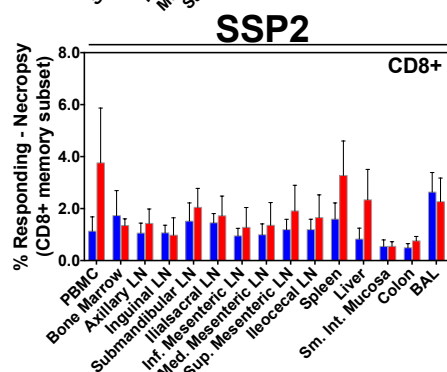

Supplement: S6 Fig — Flow cytometric ICS results of peripheral blood and tissue CD4+ and CD8+ T cell responses to the peptide mixes comprising each of the four PK antigens in 4 animals of cohort 1 (RhCMV/PK4), 3 animals of cohort 2 (ΔRh186-9/PK4) and 3 animals of control cohort 3. The average response frequencies (+SEM), corrected for memory T cells, is shown for the indicated tissues for each of the antigens. (PDF) [file pone.0210252.s006.pdf]
