# Supplementary material for: Cytomegalovirus vectors expressing Plasmodium knowlesi antigens induce immune responses that delay parasitemia upon sporozoite challenge
Source: PLoS One. 2019 Jan 23;14(1):e0210252. doi: 10.1371/journal.pone.0210252 (PMC6343944; doi:10.1371/journal.pone.0210252)
Supplement: S2 Fig — Nucleotide sequence alignment and in silico translation of the CSP insert ΔRh186-9/CSP (upper sequence) and in RhCMV/CSP (lower sequence). The sequence was generated from DNA of virus isolated from the supernatant of infected rhesus fibroblasts. The in-frame deletion in the CSP region of RhCMV/CSP resulted in an internal truncation of the repeat region. (PDF) [file pone.0210252.s002.pdf]

```
1        10        20        30        40        50        60        70        80        90
ATGAAGAACTTCATCCTGCTGGCCGTGTCCTCTATCCTGCTGGTGGACCTGCTGCCCACCCACTTCGAGCACAACGTGGACCTGTCCAGGGCCATCAAC
 M  K  N  F  I  L  L  A  V  S  S  I  L  L  V  D  L  L  P  T  H  F  E  H  N  V  D  L  S  R  A  I  N
ATGAAGAACTTCATCCTGCTGGCCGTGTCCTCTATCCTGCTGGTGGACCTGCTGCCCACCCACTTCGAGCACAACGTGGACCTGTCCAGGGCCATCAAC
 M  K  N  F  I  L  L  A  V  S  S  I  L  L  V  D  L  L  P  T  H  F  E  H  N  V  D  L  S  R  A  I  N

100       110       120       130       140       150       160       170       180       190
GTGAACGGCGTGTCCTTCAACAATGTGGACACCTCCAGCCTGGGAGCCGCCCAGGTCCGCCAGTCCGCCTCTAGAGGAAGAGGCCTGGGCGAGAAGCCC
 V  N  G  V  S  F  N  N  V  D  T  S  S  L  G  A  A  Q  V  R  Q  S  A  S  R  G  R  G  L  G  E  K  P
GTGAACGGCGTGTCCTTCAACAATGTGGACACCTCCAGCCTGGGAGCCGCCCAGGTCCGCCAGTCCGCCTCTAGAGGAAGAGGCCTGGGCGAGAAGCCC
 V  N  G  V  S  F  N  N  V  D  T  S  S  L  G  A  A  Q  V  R  Q  S  A  S  R  G  R  G  L  G  E  K  P

200       210       220       230       240       250       260       270       280       290
AAAGAGGGCGCCGACAAAGAGAAGAAGAAAGAGAAGGGTAAAGAGAAAGAGGAAGAACCCAAGAAGCCCAACGAGAACAAGCTGAAGCAGCCCAACGAG
 K  E  G  A  D  K  E  K  K  K  E  K  G  K  E  K  E  E  E  P  K  K  P  N  E  N  K  L  K  Q  P  N  E
AAAGAGGGCGCCGACAAAGAGAAGAAGAAAGAGAAGGGTAAAGAGAAAGAGGAAGAACCCAAGAAGCCCAACGAGAACAAGCTGAAGCAGCCCAACGAG
 K  E  G  A  D  K  E  K  K  K  E  K  G  K  E  K  E  E  E  P  K  K  P  N  E  N  K  L  K  Q  P  N  E

300       310       320       330       340       350       360       370       380       390
GGCCAGCCCCAGGCTCAGGGCGACGGCGCTAATGCTGGACAGCCACAGGCCCAGGGGGATGGGGCCAACGCCGGACAGCCTCAGGCTCAGGGGGATGGC
 G  Q  P  Q  A  Q  G  D  G  A  N  A  G  Q  P  Q  A  Q  G  D  G  A  N  A  G  Q  P  Q  A  Q  G  D  G
GGCCAGCCCCAGGCTCAGGGCGACGGCGCTAATGCTGGACAGCCACAGGCCCAGGGGGATGGGGCCAACGCCGGACAGCCTCAGGCTCAGGGGGATGGC
 G  Q  P  Q  A  Q  G  D  G  A  N  A  G  Q  P  Q  A  Q  G  D  G  A  N  A  G  Q  P  Q  A  Q  G  D  G

400       410       420       430       440       450       460       470       480       490
GCCAATGCTGGCCAGCCACAGGCTCAGGGGGATGGCGCTAATGCCGGGCAGCCTCAGGCTCAGGGCGACGGGGCTAACGCCGGCCAGCCACAGGCCCAG
 A  N  A  G  Q  P  Q  A  Q  G  D  G  A  N  A  G  Q  P  Q  A  Q  G  D  G  A  N  A  G  Q  P  Q  A  Q
GCCAATGCTGGCCAGCCACAGGCTCAGGGGGATGGCGCTAATGCCGGGCAGCCTCAGGCTCAGGGCGACGGGGCTAACGCCGGCCAGCCACAGGCCCAG
 A  N  A  G  Q  P  Q  A  Q  G  D  G  A  N  A  G  Q  P  Q  A  Q  G  D  G  A  N  A  G  Q  P  Q  A  Q

500       510       520       530       540       550       560       570       580       590
GGCGACGGGGCCAATGCTGGGCAGCCCCAGGCCCAGGGCGACGGCGCCAATGCCGGCCAGCCTCAGGCCCAGGGGGATGGCGCTAACGCTGGACAGCCC
 G  D  G  A  N  A  G  Q  P  Q  A  Q  G  D  G  A  N  A  G  Q  P  Q  A  Q  G  D  G  A  N  A  G  Q  P
GGCGACGGGGCCAATGCTGGGCAGCCCCAGGCCCAGGGCGACGGCGCCAATGCCGGCCAGCCTCAGGCCCAGGGGGATGGCGCTAACGCTGGACAGCCC
 G  D  G  A  N  A  G  Q  P  Q  A  Q  G  D  G  A  N  A  G  Q  P  Q  A  Q  G  D  G  A  N  A  G  Q  P

600       610       620       630       640       650       660       670       680       690
CAGGCTCAGGGGGAC------------------------------------------------------------------------------------
 Q  A  Q  G  D ------------------------------------------------------------------------------------
CAGGCTCAGGGGGACGGGGCTAACGCTGGACAGCCACAGGCTCAGGGCGACGGCGCCAACGCCGGACAGCCCCAGGCCCAGGGCGATAGAGCTAACGCC
 Q  A  Q  G  D  G  A  N  A  G  Q  P  Q  A  Q  G  D  G  A  N  A  G  Q  P  Q  A  Q  G  D  R  A  N  A

700       710       720       730       740       750       760       770       780       790
------------------------GGGGTCAATGTGCCCAGGCAGGGAAGAAACGGCGGAGGCGCCCCAGCCGGCGGAAACGAGGGAAACAAGCAGGCC
------------------------ G  V  N  V  P  R  Q  G  R  N  G  G  G  A  P  A  G  G  N  E  G  N  K  Q  A
GGGCAGCCACAGGCTCAGGGGGATGGGGCCAATGTGCCCAGGCAGGGAAGAAACGGCGGAGGCGCCCCAGCCGGCGGAAACGAGGGAAACAAGCAGGCC
 G  Q  P  Q  A  Q  G  D  G  A  N  V  P  R  Q  G  R  N  G  G  G  A  P  A  G  G  N  E  G  N  K  Q  A

800       810       820       830       840       850       860       870       880       890
GGCAAGGGCCAGGGCCAGAACAACCAGGGGGCTAATGCCCCAAACGAGAAGGTGGTGAACGACTACCTGCACAAGATCAGGTCCTCCGTGACCACCGAG
 G  K  G  Q  G  Q  N  N  Q  G  A  N  A  P  N  E  K  V  V  N  D  Y  L  H  K  I  R  S  S  V  T  T  E
GGCAAGGGCCAGGGCCAGAACAACCAGGGGGCTAATGCCCCAAACGAGAAGGTGGTGAACGACTACCTGCACAAGATCAGGTCCTCCGTGACCACCGAG
 G  K  G  Q  G  Q  N  N  Q  G  A  N  A  P  N  E  K  V  V  N  D  Y  L  H  K  I  R  S  S  V  T  T  E

900       910       920       930       940       950       960       970       980       990
TGGACCCCCTGCTCCGTGACCTGCGGCAATGGCGTGCGCATCAGAAGAAAGGCCCACGCCGGCAACAAGAAGGCCGAGGACCTGACCATGGACGACCTG
 W  T  P  C  S  V  T  C  G  N  G  V  R  I  R  R  K  A  H  A  G  N  K  K  A  E  D  L  T  M  D  D  L
TGGACCCCCTGCTCCGTGACCTGCGGCAATGGCGTGCGCATCAGAAGAAAGGCCCACGCCGGCAACAAGAAGGCCGAGGACCTGACCATGGACGACCTG
 W  T  P  C  S  V  T  C  G  N  G  V  R  I  R  R  K  A  H  A  G  N  K  K  A  E  D  L  T  M  D  D  L

1,000     1,010     1,020     1,030     1,040     1,050     1,060     1,070     1,080 1,083
GAAGTGGAAGCCTGCGTGATGGACAAGTGCGCCGGCATCTTCAACGTGGTGTCCAACTCCCTGGGCGACTACAAGGACGACGACGACAAATGA
 E  V  E  A  C  V  M  D  K  C  A  G  I  F  N  V  V  S  N  S  L  G  D  Y  K  D  D  D  D  K  *
GAAGTGGAAGCCTGCGTGATGGACAAGTGCGCCGGCATCTTCAACGTGGTGTCCAACTCCCTGGGCGACTACAAGGACGACGACGACAAATGA
 E  V  E  A  C  V  M  D  K  C  A  G  I  F  N  V  V  S  N  S  L  G  D  Y  K  D  D  D  D  K  *
```
