# Supplementary material for: Cytomegalovirus vectors expressing Plasmodium knowlesi antigens induce immune responses that delay parasitemia upon sporozoite challenge
Source: PLoS One. 2019 Jan 23;14(1):e0210252. doi: 10.1371/journal.pone.0210252 (PMC6343944; doi:10.1371/journal.pone.0210252)
Supplement: S3 Fig — (A) Comparison of T cell response magnitudes, as determined by measuring the areas under the log10 curve (AUC) of T cell frequencies for each individual RM determined by ICS, between cohort 1 (RhCMV/PK4) and Cohort 2 (ΔRh186-9/PK4) over the entire immunization period. The boxplots graph shows the average (within 95% CI) median (horizontal line), interquartile range (shaded box), and range (whiskers and outlier points) of the total T cell responses to all antigens, whereas the table shows the p-values for the comparisons of each of the antigens individually. Statistical significance was determined by Wilcoxon test and we applied the Holm p-value adjustment method for controlling the family-wise error rate over the four genes. (B) Comparison of the peak T cell response over the immunization phase either for all antigens (boxplot graph) or for each antigen individually (table). Statistical analysis was as in A). (C) Comparisons of T cell response magnitudes (AUC) determined for cohort 1 and cohort 2 after the 2nd boost. Statistical analysis was as in A). (D) Comparisons of peak T cell response magnitudes determined for cohort 1 and cohort 2 after the 2nd boost. Statistical analysis was as in A). (PDF) [file pone.0210252.s003.pdf]

A

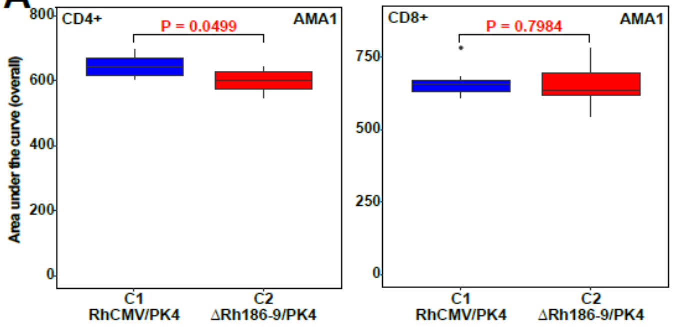

B

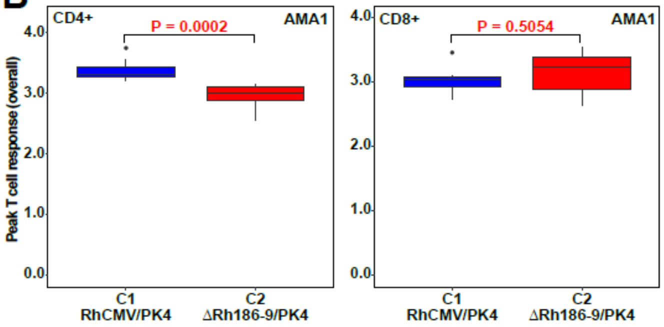

	P values Area under the curve (post-boost 2)	
Antigen	CD4+	CD8+
AMA1	0.0280	1.0000
CSP	0.0443	0.9380
MSP1c	0.0443	1.0000
SSP2	0.0443	1.0000

	P values Area under the curve (post-boost 2)	
Antigen	CD4+	CD8+
AMA1	0.0037	1.0000
CSP	0.0037	0.9380
MSP1c	0.0037	1.0000
SSP2	0.1304	1.0000

C

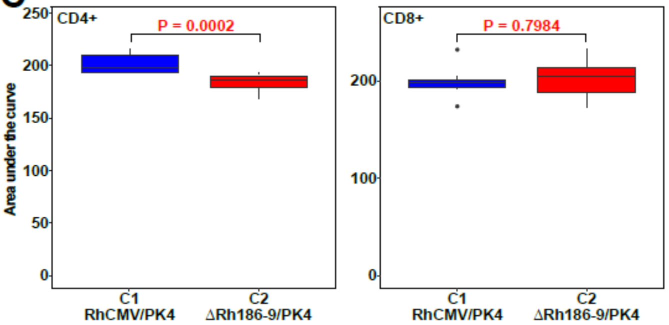

D

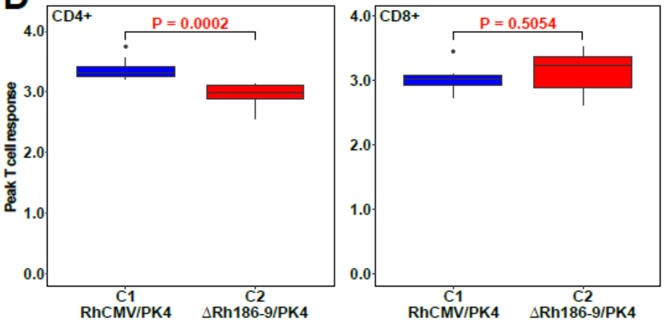

	P values Area under the curve (post-boost 2)	
Antigen	CD4+	CD8+
AMA1	0.0012	0.9846
CSP	0.0006	0.5215
MSP1c	0.0009	1.0000
SSP2	0.0104	1.0000

	P values Area under the curve (post-boost 2)	
Antigen	CD4+	CD8+
AMA1	0.0037	1.0000
CSP	0.0037	0.9380
MSP1c	0.0037	1.0000
SSP2	0.1304	1.0000
