# Supplementary material for: Cytomegalovirus vectors expressing Plasmodium knowlesi antigens induce immune responses that delay parasitemia upon sporozoite challenge
Source: PLoS One. 2019 Jan 23;14(1):e0210252. doi: 10.1371/journal.pone.0210252 (PMC6343944; doi:10.1371/journal.pone.0210252)
Supplement: S5 Fig — Parasitemia was determined as described in the Materials and Methods. Animals were treated with anti-malarial drugs when parasites exceeded 2% parasitemia (>400 infected RBC) on the indicated days. (PDF) [file pone.0210252.s005.pdf]

Group	Animal ID	Day 6	Day 7	Day 8	Day 9	Day 10	Day 11	Day 12	Day 13
C1: RhCMV/PK4	C1-1	0	0	0	0	4	59	670	Treated
C1: RhCMV/PK4	C1-2	0	0	1	16	62	621	Treated	Treated
C1: RhCMV/PK4	C1-3	0	0	0	1	32	243	2029	Treated
C1: RhCMV/PK4	C1-4	0	0	0	1	4	38	1767	Treated
C1: RhCMV/PK4	C1-5	0	0	0	3	23	250	1100	Treated
C1: RhCMV/PK4	C1-6	0	0	0	5	25	204	3533	Treated
C1: RhCMV/PK4	C1-7	0	0	0	3	19	107	2430	Treated
C1: RhCMV/PK4	C1-8	0	0	0	5	77	1222	Treated	Treated
C2: ΔRh186-9/PK4	C2-1	0	0	0	3	36	260	564	Treated
C2: ΔRh186-9/PK4	C2-2	0	0	0	3	38	326	669	Treated
C2: ΔRh186-9/PK4	C2-3	0	0	0	13	48	761	Treated	Treated
C2: ΔRh186-9/PK4	C2-4	0	0	0	4	50	598	Treated	Treated
C2: ΔRh186-9/PK4	C2-5	0	0	0	0	9	87	1664	Treated
C2: ΔRh186-9/PK4	C2-6	0	0	0	7	43	941	Treated	Treated
C2: ΔRh186-9/PK4	C2-7	0	0	0	0	0	0	0	0
C2: ΔRh186-9/PK4	C2-8	0	0	0	6	50	314	Treated	Treated
C3: Unvaccinated	C3-1	0	0	0	13	134	1457	Treated	Treated
C3: Unvaccinated	C3-2	0	0	1	5	27	490	Treated	Treated
C3: Unvaccinated	C3-3	0	0	1	20	133	2290	Treated	Treated
C3: Unvaccinated	C3-4	0	0	1	11	95	1600	Treated	Treated
C3: Unvaccinated	C3-5	0	0	1	16	263	2100	Treated	Treated
C3: Unvaccinated	C3-6	0	0	1	5	29	256	1567	Treated
C3: Unvaccinated	C3-7	0	0	0	8	19	244	4533	Treated
C3: Unvaccinated	C3-8	0	0	0	11	158	1156	Treated	Treated
